# Periodically Disturbing the Spatial Structure of Biofilms Can Affect the Production of an Essential Virulence Factor in *Pseudomonas aeruginosa*

**DOI:** 10.1128/mSystems.00961-21

**Published:** 2021-09-28

**Authors:** Rebecca J. Quinn, Ivana Barraza, Laura García-Diéguez, Camryn Pajon, Lauren E. Krausfeldt, Kerollos Ibrahim, Laura A. Enzinna, Morgan E. Thorn, Omar Tonsi Eldakar, Travis J. A. Craddock, Robert P. Smith

**Affiliations:** a Department of Biological Sciences, Halmos College of Arts and Science, Nova Southeastern University, Fort Lauderdale, Florida, USA; b Clinical Systems Biology Group, Institute for Neuro-Immune Medicine, Nova Southeastern University, Fort Lauderdale, Florida, USA; c Department of Psychology and Neuroscience, College of Psychology, Nova Southeastern University, Fort Lauderdale, Florida, USA; d Department of Computer Science, College of Engineering and Computing, Nova Southeastern University, Fort Lauderdale, Florida, USA; e Department of Clinical Immunology, College of Osteopathic Medicine, Nova Southeastern University, Fort Lauderdale, Florida, USA; University of Delhi

**Keywords:** *Pseudomonas aeruginosa*, pyoverdine, biofilms, disturbance, mathematical modeling, virulence factors

## Abstract

Understanding the environmental factors that affect the production of virulence factors has major implications in evolution and medicine. While spatial structure is important in virulence factor production, observations of this relationship have occurred in undisturbed or continuously disturbed environments. However, natural environments are subject to periodic fluctuations, including changes in physical forces, which could alter the spatial structure of bacterial populations and impact virulence factor production. Using Pseudomonas aeruginosa PA14, we periodically applied a physical force to biofilms and examined production of pyoverdine. Intermediate frequencies of disturbance reduced the amount of pyoverdine produced compared to undisturbed or frequently disturbed conditions. To explore the generality of this finding, we examined how an intermediate disturbance frequency affected pyoverdine production in 21 different strains of P. aeruginosa. Periodic disturbance increased, decreased, or did not change the amount of pyoverdine produced relative to undisturbed populations. Mathematical modeling predicts that interactions between pyoverdine synthesis rate and biofilm density determine the amount of pyoverdine synthesized. When the pyoverdine synthesis rates are high, depletion of the biofilm due to disturbance reduces the accumulation of pyoverdine. At intermediate synthesis rates, production of pyoverdine increases during disturbance as bacteria dispersed into the planktonic state enjoy increased growth and pyoverdine production rates. At low synthesis rates, disturbance does not alter the amount of pyoverdine produced since disturbance-driven access to nutrients does not augment pyoverdine synthesis. Our results suggest that environmental conditions shape robustness in the production of virulence factors and may lead to novel approaches to treat infections.

**IMPORTANCE** Virulence factors are required to cause infections. Previous work has shown that the spatial organization of a population, such as a biofilm, can increase the production of some virulence factors, including pyoverdine, which is produced by Pseudomonas aeruginosa. Pyoverdine is essential for the infection process, and reducing its production can limit infections. We have discovered that periodically changing the spatial structure of a biofilm of P. aeruginosa strain PA14 using a physical force can reduce the production of pyoverdine. A mathematical model suggests that this is due to the disruption of spatial organization. Using additional strains of P. aeruginosa isolated from patients and the environment, we use experiments and modeling to show that this reduction in pyoverdine is due to interactions between biofilm density and the synthesis rate of pyoverdine. Our results identify conditions where pyoverdine production is reduced and may lead to novel ways to treat infections.

## INTRODUCTION

Virulence factors enable bacteria to colonize hosts, cause disease, and interfere with immune systems (1). They are critical components of pathogenesis since their removal via genetic means can prevent infection (2). While the genetic underpinnings of virulence factor expression have been identified for numerous pathogens (3), less is known about how fluctuations in the growth environment affect the production of virulence factors. This is critical to understand; pathogens sense, respond, and adapt to the host growth environment (4, 5), and fluctuations can provide advantages to either the host or the pathogen (6). Understanding how fluctuations in the environment affect the production of virulence factors has implications in microbial ecology (7), evolution (8, 9), and medicine (10).

The spatial structure of a bacterial population is a key determinant of virulence factor production (11). The formation of spatially organized populations (e.g., biofilms) can lead to the enhanced production of virulence factors (see Table S1). For example, small diffusible molecules, such as those involved in quorum sensing, can accumulate inside biofilms, which can augment expression of downstream elements, including virulence factors (12). Alternatively, some virulence factors show increased expression when bacteria are in the planktonic state where spatial structure is minimized (13). Our understanding of how spatial structure influences virulence factor production is largely based upon studies that are performed in constantly disturbed (e.g., well-mixed tubes) or stationary conditions (e.g., agar plates). These conditions do not necessarily mimic natural environments where environmental conditions will fluctuate. One type of fluctuation that is important in perturbing spatial structure are changes in physical forces (14, 15). Changes in fluid flow can be observed in aquatic environments (16), in medical devices (17), and in hosts (18). These can change or abolish biofilm structure (19, 20). Vibrations, frictional forces, and additional mechanical stressors can impact surface attachment and biofilm morphology (21, 22). In a natural environment, these forces are not continuous; they can fluctuate or occur periodically. Both soils (23) and tissues (24) can experience periodic changes in vibrational forces and aqueous environments can experience rapid changes in fluid flow, which affect shear forces (25). If a periodic change in force is sufficiently strong, it can lead to change in the spatial structure of a bacterial population, which may impact virulence factor production. While plausible, this relationship remains unstudied. Discovering how periodic fluctuations in physical forces affect virulence factor production can help determine evolutionary constraints that shape virulence factor production and how the robustness of such systems change across growth environments. This may lead to strategies to attenuate virulence factor production.

10.1128/mSystems.00961-21.8 TABLE S1 Examples of pathogens and virulence factors whose expression changes as a result of spatial structure. Download Table S1, DOCX file, 0.01 MB. Copyright © 2021 Quinn et al. 2021 Quinn et al. https://creativecommons.org/licenses/by/4.0/ This content is distributed under the terms of the Creative Commons Attribution 4.0 International license.

In this study, we used experimentation and mathematical modeling to determine how disturbing the spatial structure of biofilms composed of Pseudomonas aeruginosa with periodically applied physical forces alters the production of pyoverdine. We studied P. aeruginosa since it can cause infections in a variety of tissues (26–28). Environments where P. aeruginosa inhabits (soils [29], the trachea [18] and catheters [30]) are subjected to periodic fluctuations in physical forces. We studied the virulence factor pyoverdine since it has a well-described production pathway (31) and is essential for biofilm formation (32) and virulence (33). The degree of spatial structure of the population influences the amount of pyoverdine produced; aggregates of bacteria show increased pyoverdine synthesis (34), cell-cell contacts can limit pyoverdine diffusion (35), and bacteria in the planktonic state show augmented pyoverdine synthesis relative to their biofilm state counterparts (13, 36). Pyoverdine has a well-described role in pathogenesis; after being secreted from the cell, it sequesters iron from the environment and then returns the sequestered iron to the bacteria. This augments growth, the production of pyoverdine, and the expression of additional virulence factors (31).

## RESULTS

### An experimental approach to disturbing the spatial structure of biofilms.

**FIG 1:**
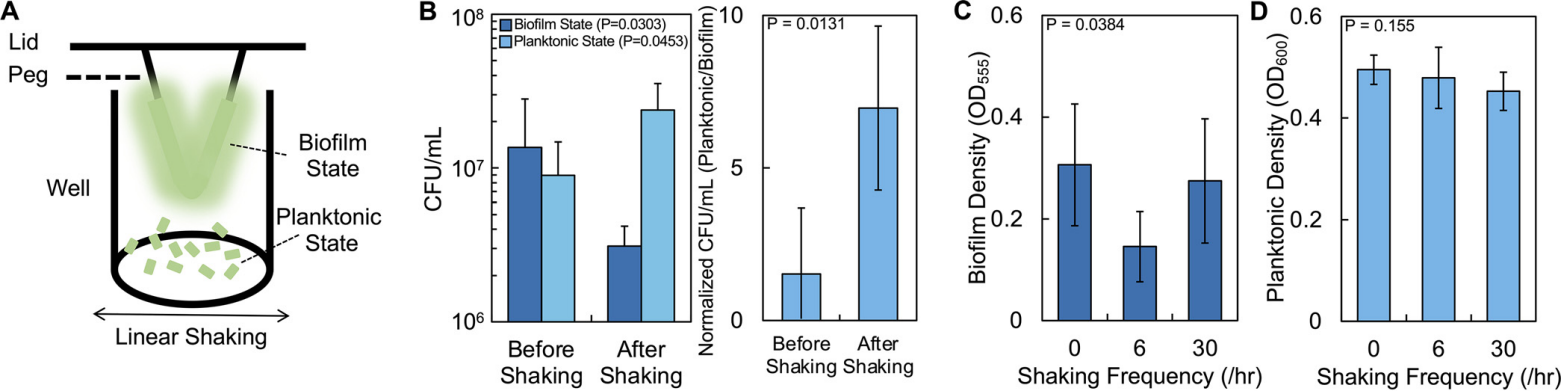
An experimental approach to periodically applying a physical force to structured bacterial populations. (A) We used the Innovotech MBEC Biofilm Inoculator device to grow biofilms. After growing biofilms, we used the linear shaking function of a plate reader to disturb the distribution of bacteria in the biofilm. (B) Density of bacteria in the biofilm and planktonic states before and after a single shaking event. (Left panel) Absolute cell density (biofilm, *P = *0.0303, planktonic, *P = *0.0453, Mann-Whitney [*P* = 0.0006, Shapiro-Wilk]). (Right panel) Planktonic cell density normalized by biofilm cell density (*P = *0.0131, Mann-Whitney [*P* = 0.0191, Shapiro-Wilk]). Standard deviations from six biological replicates were determined. (C) Average biofilm density of strain PA14 at 24 h after shaking at the disturbance frequency indicated (*P = *0.0384, one-way analysis of variance [ANOVA]). For panels C and D, standard deviations from a minimum of four biological replicates were determined. (D) Average density of bacteria in the planktonic state at 24 h after shaking at the disturbance frequency indicated (*P = *0.155, one-way ANOVA).

To periodically disturb the spatial structure of biofilms using physical force, we used an MBEC biofilm inoculator device (Fig. 1A) (37). This system consists of a 96-well plate with rounded plastic pegs adhered to the inside of the lid. Each peg sits inside a well of a 96-well plate that contains liquid medium. Biofilms form on the peg, while bacteria in the planktonic state grow in the medium surrounding the peg. The density of bacteria in the biofilm and planktonic states can be measured using a crystal violet assay quantified at an optical density at 555 nm (OD_555_) (38) and OD_600_, respectively.

Our previous work demonstrated that we could use the linear shaking function of a microplate reader to perturb the spatial structure of bacteria embedded in soft agar (39). While the forces that P. aeruginosa can encounter in the environment are diverse, linear shaking captures the general effect of such forces: the ability to periodically alter the positions of bacteria. Physical forces encountered in natural environments, including changes in fluid flow (40) and additional mechanical forces (41), can transition bacteria between the biofilm and planktonic states. To determine whether we could use linear shaking to disrupt the spatial distribution of bacteria grown in the MBEC device, we grew biofilms composed of P. aeruginosa strain PA14 and quantified the density of bacteria in the biofilm and planktonic states before and after a single shaking event (Fig. 1B). We observed a significant increase in the density of bacteria in the planktonic state after a single shaking event, which coincided with a significant decrease in the density of bacteria in the biofilm state.

To determine how multiple shaking events over 24 h affected the distribution of bacteria in the biofilm and planktonic states, we grew biofilms of strain PA14 and subjected them to periodic shaking at three frequencies: 0 shakes/hour (0/h), 6/h, and 30/h. The 0/h and 30/h shakes served as the extremes of disturbance in our experiments; 0/h represented an undisturbed condition, and 30/h represented a frequently disturbed condition. 6/h served as an intermediate shaking frequency between these extremes. We observed a biphasic relationship between biofilm density and shaking frequency; biofilm density decreased at 6/h (relative to 0/h) but increased at 30/h (Fig. 1C). This increase at a high shaking frequency (30/h) is consistent with work showing that increasing shear force can promote biofilm formation (42, 43). In contrast, we observed a small, but insignificant, decrease in the density of bacteria in the planktonic state with increasing shaking frequency (Fig. 1D). Overall, using the linear shaking function of a plate reader could disrupt the spatial distribution of biofilms composed of P. aeruginosa.

### Periodically disturbing the spatial structure of biofilms can alter the amount of pyoverdine.

**FIG 2:**
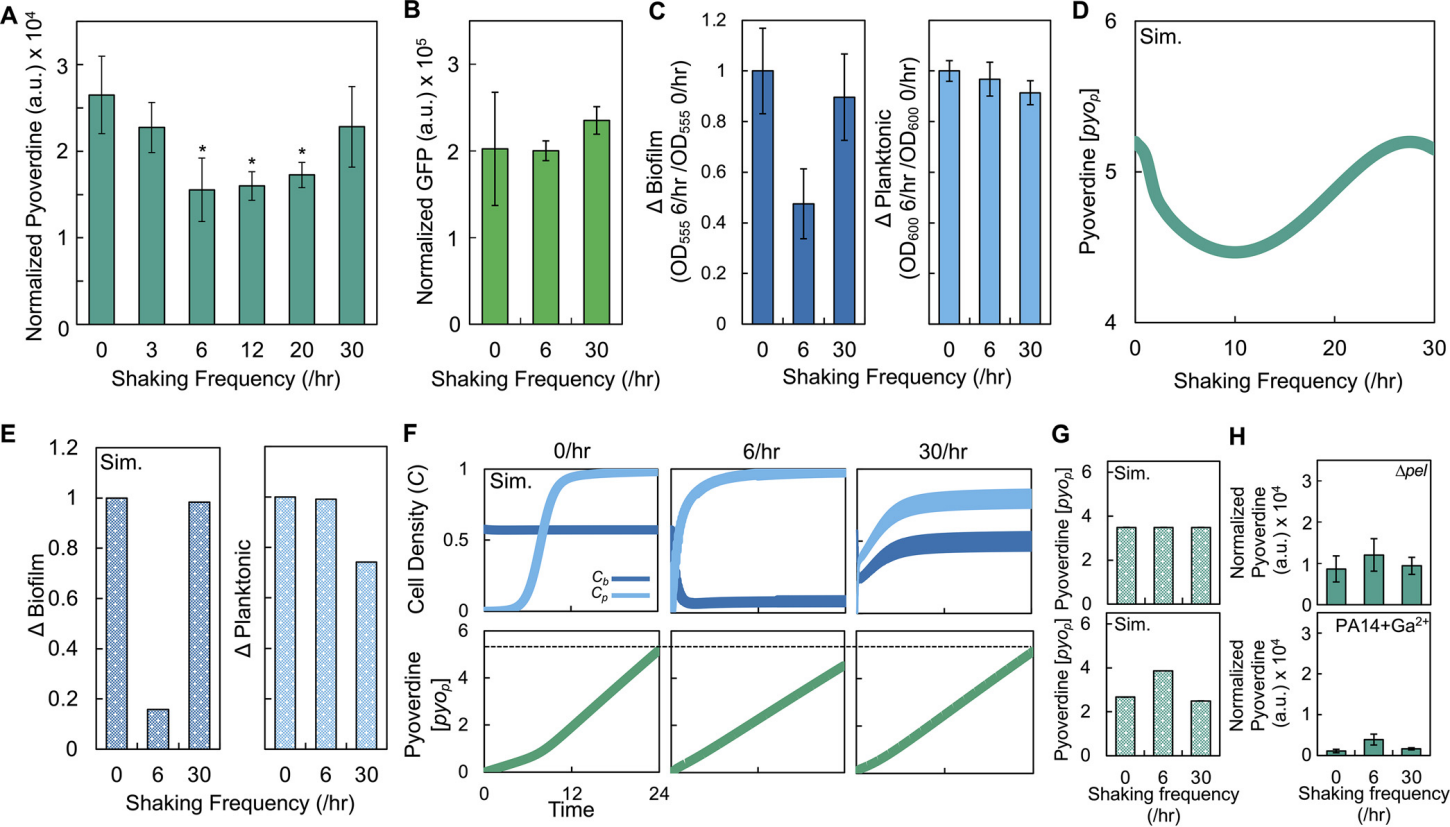
Periodically disturbing biofilms of P. aeruginosa strain PA14 can reduce the amount of pyoverdine. (A) Amount of pyoverdine in growth medium with 0% glucose after periodically disturbing biofilms composed of P. aeruginosa strain PA14 for 24 h. Pyoverdine normalized by cell density (OD_600_). *, Significant differences between 0/h and 30/h (*P < *0.016, two-tailed *t* test). For all panels, standard deviations from a minimum of three biological replicates were determined. The amount of pyoverdine is greater in the medium surrounding the peg compared to pyoverdine quantified from bacteria in the biofilm. Thus, we are measuring the majority of pyoverdine in our experiments (see Fig. S1A). We confirmed that we are measuring pyoverdine using a strain of PA14 that lacks the ability to make pyoverdine (Δ*pvd*; Fig. S1B) and a strain that lacks the ability to make pyocyanin (Δ*phz1* Δ*phz2*; Fig. S1C). (B) Amount of GFP produced by P. aeruginosa as a function of disturbance frequency. GFP (a.u.) was normalized by OD_600_. There was a significant increase in GFP at 30/h relative to the 6/h condition (*P = *0.012, two-tailed *t* test). Differences were not observed between 0/h and 6/h or 30/h (*P ≥ *0.21, two-tailed *t* test). (C) Change in the density of bacteria in the biofilm (Δbiofilm, left panel, *P = *0.0384, one-way ANOVA) and planktonic (Δplanktonic, right panel, *P* = 0.155, one-way ANOVA) states due to periodic disturbance at 6/h and 30/h. (D) Simulations using equations 5 to 8 showing the effect of disturbance on pyoverdine synthesis. *t *=* *24 h for panels D to G. The parameters are presented in Table S3. Sensitivity analysis results are shown in Fig. S1F. For panels D to G, Sim. = simulation results. (E) Simulated Δbiofilm and Δplanktonic values. (F) Simulations showing the temporal changes in the density of bacteria in the biofilm and planktonic states (top panels) and the amount of pyoverdine (bottom panels). The dotted line facilitates comparison between plots. (G) Simulations showing the effect of removing biofilm structure (top, *C_b_*, [*pyo_b_*] and [*pyo_p_*] = 0, *C_p_* = 0.1) or reducing pyoverdine functionality (bottom, δ increased to 0.21 [from 0.11]). (H) Experiments showing the effect of removing biofilm structure (Δ*pel* strain, top, *P = *0.11 [Kruskal-Wallis] and *P* = 0.0011 [Shapiro-Wilk]) or reducing pyoverdine functionality by adding 10 μM gallium to the medium (bottom, *P < *0.0001 [Kruskal-Wallis] and *P < *0.0001 [Shapiro-Wilk]).

We next sought to examine how periodic disturbance would affect pyoverdine production. We grew biofilms of P. aeruginosa PA14 for 24 h, placed the biofilms in fresh medium and shook the biofilms at different frequencies. After 24 h, we quantified the amount of pyoverdine in the planktonic state and normalized this value by cell density (OD_600_). At disturbance frequencies of 6/h, 12/h, and 20/h, there was a significant reduction in pyoverdine relative to 0/h and 30/h (Fig. 2A). When biofilms composed of green fluorescent protein (GFP)-expressing P. aeruginosa were disturbed under the same conditions, GFP normalized by cell density (OD_600_) did not show a decrease at 6/h (Fig. 2B). This indicated that the reduction of pyoverdine was not due to perturbations to gene expression.

10.1128/mSystems.00961-21.1 FIG S1 Quantification of pyoverdine and sensitivity analysis. (A) Quantity of pyoverdine in liquid medium (well, planktonic state) and in cells growing in the biofilm (peg, biofilm state). For panels A to C, pyoverdine normalized based on the OD_600_. *P = *0.008, two-tailed *t* test. Standard deviations from a minimum of four biological replicates are shown. (B) Amount of pyoverdine quantified from wild-type PA14 and a strain of PA14 that lacked the ability to synthesize pyoverdine (*Δpvd*). Glucose = 0%. Shaking frequency = 0/h. (Inset) Cell densities of wild-type PA14 and *Δpvd* strains. Standard deviations from a minimum of five biological replicates are shown. For both plots, *P < *0.0001, two-tailed *t* test. (C) Amount of pyoverdine quantified from wild-type PA14 and a strain of PA14 that lacks the ability to synthesize pyocyanin (*Δphz1 Δphz2*). Glucose = 0%. Shaking frequency = 0/h. There was no significant difference in the amounts of pyoverdine produced between wild-type and *Δphz1 Δphz2* strains, indicating that our pyoverdine measurement assay is not detecting pyocyanin (*P = *0.33, two-tailed *t* test). (Inset) Cell density of wild-type PA14 and *Δphz1 Δphz2*. *P = *0.015, two-tailed t-test. Standard deviations from a minimum of eight biological replicates were determined. (D) Expression of *pvdA* as a function of shaking frequency. We measured *pvdA* expression in the 0/h, 6/h, and 30/h conditions after 24 h in medium containing 0, 1, and 2% glucose. When comparing all expression levels across shaking frequencies and percentage of glucose, *P = *0.0909 (one-way ANOVA). Standard deviations from four biological replicates were determined. (E) Amount of pyoverdine (arbitrary units, a.u.) diffused from biofilms of PA14 to the surrounding medium (1× PBS) over 1 h. To reduce bacterial dispersal and growth, biofilms were washed in 1× PBS to remove nonadhered cells prior to placing bacteria in PBS. The use of PBS serves to restrict bacterial growth. The surrounding medium was filtered to remove bacteria prior to quantification of the pyoverdine. Standard deviations from three biological replicates each consisting of three technical replicates were determined. *P = *0.052, two-tailed *t* test. (F) Sensitivity analysis of select parameters in our model (equations 5 to 8). The parameter varied is indicated in the bottom left. The solid line indicates simulations performed with the baseline parameter set in Table S3. Sim. = simulation results. Download FIG S1, JPG file, 0.5 MB. Copyright © 2021 Quinn et al. 2021 Quinn et al. https://creativecommons.org/licenses/by/4.0/ This content is distributed under the terms of the Creative Commons Attribution 4.0 International license.

10.1128/mSystems.00961-21.10 TABLE S3 Parameters used in our model (see equations 5 to 8 in Materials and Methods). Download Table S3, DOCX file, 0.02 MB. Copyright © 2021 Quinn et al. 2021 Quinn et al. https://creativecommons.org/licenses/by/4.0/ This content is distributed under the terms of the Creative Commons Attribution 4.0 International license.

To understand how periodic spatial disturbance over 24 h affected the distribution of bacteria compared to 0/h, we determined the relative change in the density of bacteria in the biofilm and planktonic states, which we call “Δbiofilm” and “Δplanktonic,” respectively. To calculate Δbiofilm, we used crystal violet to measure the density of the biofilm after 24 h of disturbance. We then normalized each value by the density of the biofilm observed at 0/h. To calculate Δplanktonic, we used OD_600_ to measure the density of bacteria in the planktonic state after 24 h of disturbance. As described above, we normalized each value by the density of bacteria in the planktonic state observed at 0/h. We found that Δbiofilm was reduced at 6/h but remained relatively unchanged at 30/h compared to 0/h (Fig. 2C). Δplanktonic was insignificantly reduced at 30/h relative to 0/h, while at 6/h it was nearly indistinguishable from the 0/h value (Fig. 2C). This indicated that periodic disturbance reduced biofilm density at 6/h but had no significant influence on the density of bacteria in the planktonic state.

### A reduction in biofilm density reduces pyoverdine synthesis at intermediate shaking frequency.

One challenge with our experimental setup was the inability to measure the change in the distribution of bacteria and the amount of pyoverdine in real time or at multiple points during an experiment. This was owing to limitations of the MBEC device, where the peg serves to obscure the measurement of bacteria and pyoverdine without removing the lid. Removal of the lid during an experiment would alter the biofilm structure itself, which would confound our results. To overcome this challenge, we created a mathematical model that considers growth of bacteria and pyoverdine production of two distinct populations in the biofilm and planktonic states (see equations 5 to 8 in Materials and Methods). As in our experiments, we consider a population of bacteria in the biofilm state that have been placed in fresh medium (initially without bacteria in the planktonic state). Pyoverdine is synthesized by both populations according to first order kinetics and is dependent upon cell density. As previously observed (36), bacteria in the planktonic state have a higher pyoverdine synthesis rate relative to their biofilm state counterparts. However, as supported through RT-qPCR of *pvdA* (see Fig. S1 in the supplemental material), our model does not assume shaking frequency-dependent changes in the expression of genes involved in the synthesis of pyoverdine. That is, while bacteria in the planktonic state have increased pyoverdine synthesis owing to their positioning in the environment, this synthesis rate is consistent among all shaking frequencies. Bacteria grow in both states according to logistic growth that is scaled by the amount of pyoverdine in each state; increasing the amount of pyoverdine serves to increase the growth rate. This is consistent with previous work showing that removing pyoverdine synthesis through gene deletion (33) or attenuation of pyoverdine functionality (44) reduces the growth rate. In addition, bacteria in the planktonic state have a greater growth rate owing to additional nutrient access (45). Pyoverdine and bacteria in the biofilm state can diffuse to the planktonic state; the former was confirmed using a diffusion assay (see Fig. S1). Periodic disturbance transitions bacteria and pyoverdine into the planktonic state above the rate of diffusion. Bacteria can also transition from the planktonic state to the biofilm state in a shaking dependent fashion; as shaking increases, the amount of bacteria entering the biofilm state increases. This is consistent with studies that have demonstrated that increasing shear force promotes biofilm formation (42, 43). Model development and parameter estimation can be found in Materials and Methods. Parameters are presented in Table S3.

Our model predicts that the amount of pyoverdine is reduced at intermediate, but not high, shaking frequencies (Fig. 2D). Moreover, our model predicts the experimentally observed changes in Δbiofilm and Δplanktonic; Δbiofilm is reduced at 6/h, and Δplanktonic is reduced at 30/h (Fig. 2E). To understand the mechanism that led to a decrease in the amount of pyoverdine at 6/h, but not at 30/h, we simulated the temporal changes in the density of bacteria in the biofilm and planktonic states and the amount of pyoverdine (Fig. 2F). Our simulations predict that at 0/h, bacteria transition from the biofilm state into the planktonic state by diffusion, resulting in a high population density in both states after 24 h. The total amount of pyoverdine produced is a sum of pyoverdine synthesized by bacteria in biofilm state, which diffuses to the surrounding medium, and pyoverdine synthesized by bacteria in the planktonic state. At 6/h, the density of bacteria in the biofilm state is reduced owing to periodic disturbance. The rapid and sustained reduction in the density of bacteria in the biofilm state reduces the amount of pyoverdine that can diffuse from the biofilm into the surrounding medium. While there is an early increase in the number of bacteria in the planktonic state, the amount of pyoverdine produced by these bacteria is insufficient to compensate for the amount of pyoverdine lost due to the reduction in biofilm density. Accordingly, the total amount of pyoverdine produced by both populations is reduced compared to 0/h. At 30/h, frequent disturbance rapidly removes bacteria from the biofilm state into the planktonic state. However, as increased shear force promotes the formation of the biofilm, biofilm density is quickly restored. The combined effort of high-density populations in the biofilm, which contributes pyoverdine to the surrounding medium via diffusion, and planktonic states synthesizes sufficient pyoverdine such that there is no difference between 0/h and 30/h.

Since our model predicts that the change in biofilm density is critical to changes in the amount of pyoverdine, we simulated the effect of removing biofilm structure. We initialized our simulations with a small population of bacteria in the planktonic state, without bacteria in the biofilm state, and removed the ability of the bacteria to transition from the planktonic state to the biofilm state. Our model predicts that the amount of pyoverdine during periodic disturbance does not change relative to 0/h (Fig. 2G, top). To test these predictions, we used a strain of P. aeruginosa that does not form biofilms (Δ*pel*) (46); periodic disturbance at 6/h and 30/h did not alter the amount of pyoverdine relative to 0/h (Fig. 2H, top). These results are consistent with our data (see Fig. S1) showing that the expression level of genes involved in pyoverdine synthesis do not change as a function of disturbance frequency. Otherwise, a reduction in the amount of pyoverdine at 6/h would have been observed. To examine how attenuating the functionality of pyoverdine would impact the effect of periodic disturbance on the amount of pyoverdine produced, we increased the value of δ in our model, which represents the maximal rate at which growth is reduced owing to lack of iron uptake via pyoverdine. Increasing δ would effectively decrease the functionality of pyoverdine; more pyoverdine would be required to augment growth relative to smaller values of δ. Our model predicts that the amount of pyoverdine increases at 6/h relative to 0/h and 30/h (Fig. 2G, bottom). To test this prediction, we disturbed biofilms of PA14 in the presence of gallium nitrate, which reduces pyoverdine functionality (44). We observed a significant increase in pyoverdine at 6/h, relative to 0/h and 30/h (Fig. 2H, bottom). This analysis provides support of our modeling predictions and shows that the presence of a biofilm and pyoverdine functionality are required to observe a decrease in pyoverdine at intermediate disturbance frequencies.

### The reduction in pyoverdine at 6/h is consistent when biofilm density, pyoverdine synthesis, and growth rate are perturbed.

**FIG 3:**
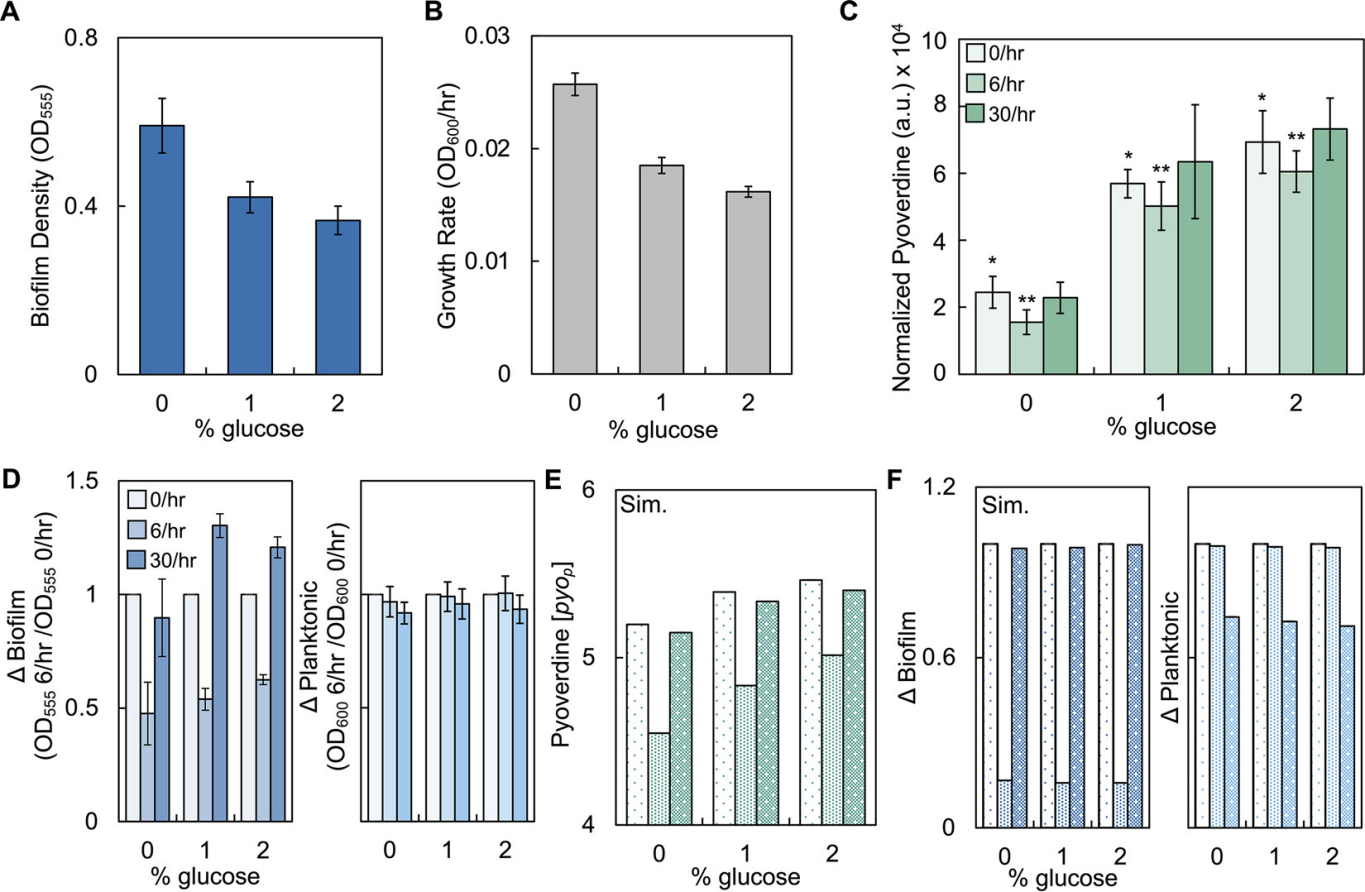
The decrease in pyoverdine is maintained when the initial biofilm density, growth rate, and pyoverdine synthesis are simultaneously perturbed using glucose. (A) Average initial biofilm density of P. aeruginosa grown in media with increasing percentages of glucose. *P < *0.0001, one-way ANOVA. Standard deviations from five biological replicates were determined. (B) Average growth rate of P. aeruginosa in media with increasing glucose. *P < *0.001, one-way ANOVA. Standard deviations from three biological replicates were determined. (C) Amount of pyoverdine in growth media with increasing percentages of glucose after periodically disturbing biofilms. Pyoverdine normalized by cell density (OD_600_). *, Statistical difference between undisturbed condition across percentages of glucose (*P < *0.0001, Kruskal-Wallis, Shapiro-Wilk, *P = *0.002); **, statistical difference between undisturbed (0/h) and 30/h conditions (*P ≤ *0.0317, Mann-Whitney, all conditions, Shapiro-Wilk, *P < *0.0001). (D) Change in the density of bacteria in biofilm (Δbiofilm, left panel, *P ≤ *0.0384 for all % glucose, one-way ANOVA) and planktonic (Δplanktonic, right panel, *P ≥ *0.155 for all % glucose, one-way ANOVA) states due to periodic disturbance at 6/h and 30/h. Standard deviations from a minimum of four biological replicates were determined. (E) Simulations showing the effect of changing growth rate (*μ*), initial biofilm density (*C_b_*), and pyoverdine synthesis rate (*k_s_*) on the amount of pyoverdine produced during periodic disturbance. For panels E and F, *t *=* *24 h. Parameters are presented in Table S3. For all panels, Sim. = simulations results. (F) Simulated Δbiofilm and Δplanktonic values for the conditions depicted in panels E.

Previous work has indicated that carbon sources can influence biofilm density, growth rate, and the amount of pyoverdine produced by pseudomonads (47–49). To determine how changes to these variables would affect the ability of disturbance to reduce pyoverdine at 6/h, we increased the percentage of glucose in the growth medium. This served to decrease biofilm density (Fig. 3A) and growth rate (Fig. 3B) but increased pyoverdine synthesis (Fig. 3C). The decrease in biofilm density with increasing glucose differs from a previous study that indicated glucose increases biofilm density (49). Quantification of biofilm density in the absence of the MBEC device confirmed these previous results (see Fig. S2). Next, we periodically disturbed biofilms formed in media with increasing glucose. We observed that the amount of pyoverdine decreased at 6/h (relative to 0/h and 30/h) when the percentage of glucose in the medium was increased. Quantification of Δbiofilm and Δplanktonic showed similar results to when 0% glucose was used in the medium; Δbiofilm showed a biphasic relationship with shaking frequency (Fig. 3D, left), whereas Δplanktonic was slightly reduced at 30/h (Fig. 3D, right). Our model predictions were consistent with these findings. To capture the effect of increasing the concentration of glucose in the medium, we simultaneously decreased growth rate and initial biofilm density, while increasing pyoverdine production rate (parameters in Table S3). Our model predicts a reduction in pyoverdine amount at 6/h (relative to 0/h and 30/h, Fig. 3E), that Δbiofilm is reduced at 6/h, and that Δplanktonic decreases at 30/h (Fig. 3F). Our combined experimental and modeling analysis show that the reduction in the amount of pyoverdine at 6/h is observable under conditions that alter initial biofilm density, growth rate, and pyoverdine synthesis rate.

10.1128/mSystems.00961-21.2 FIG S2 Growth characteristics of P. aeruginosa strains used in this study. (A) Initial density of biofilms of different strains of P. aeruginosa as measured using crystal violet (OD_555_). Biofilms were grown for a total of 24 h. Periodic disturbance was not applied. Standard deviations from three biological replicates were determined. (B) Average growth rate of P. aeruginosa strains measured using the OD_600_ in medium without glucose. Standard deviations from three biological replicates were determined. (C) Average amount of pyoverdine produced in the undisturbed condition (0/h) for strains grown in medium without glucose. Standard deviations from six biological replicates were determined. (D) Linear correlations between growth characteristics of P. aeruginosa strains used in this study. We did not find a significant linear correlation between initial biofilm density and growth rate (linear regression, *P = *0.3736), normalized pyoverdine (0/h) and growth rate (linear regression, *P = *0.859), and initial biofilm density and normalized pyoverdine (0/h, linear regression, *P = *0.2947). Data points drawn from strains grown in all three growth media (0, 1, and 2% glucose). (E) Biofilm density of select strains in medium with 0 and 2% glucose grown in the absence of the MBEC plate. Increasing glucose increased biofilm density (*P = *0.0012, Mann-Whitney, *P < *0.0001, Shapiro-Wilk). Download FIG S2, JPG file, 0.6 MB. Copyright © 2021 Quinn et al. 2021 Quinn et al. https://creativecommons.org/licenses/by/4.0/ This content is distributed under the terms of the Creative Commons Attribution 4.0 International license.

### Periodic disturbance can increase, decrease or leave the amount of pyoverdine unchanged.

Environmental factors, such as pH and community diversity, can affect strain specific pyoverdine production rates (50). To examine how strains isolated from diverse environments would be impacted by periodic disturbance, we acquired 20 additional strains of P. aeruginosa that were isolated from the environment and the clinic (see Table S2). These strains varied in initial biofilm density, growth rate, and the amount of pyoverdine produced in the 0/h condition, but none of these characteristics were linearly correlated with each other (see Fig. S2). Thus, each strain represented a unique combination of growth rate, biofilm density, and pyoverdine synthesis. Using this set of strains, we could develop an understanding of the key facets of bacterial physiology that allowed pyoverdine to be reduced at 6/h. We chose to study 6/h since it resulted in the greatest reduction in pyoverdine synthesis in PA14 and was between the extreme conditions of disturbance (0/h and 30/h).

10.1128/mSystems.00961-21.9 TABLE S2 Strains used in this study. Download Table S2, DOCX file, 0.01 MB. Copyright © 2021 Quinn et al. 2021 Quinn et al. https://creativecommons.org/licenses/by/4.0/ This content is distributed under the terms of the Creative Commons Attribution 4.0 International license.

**FIG 4:**
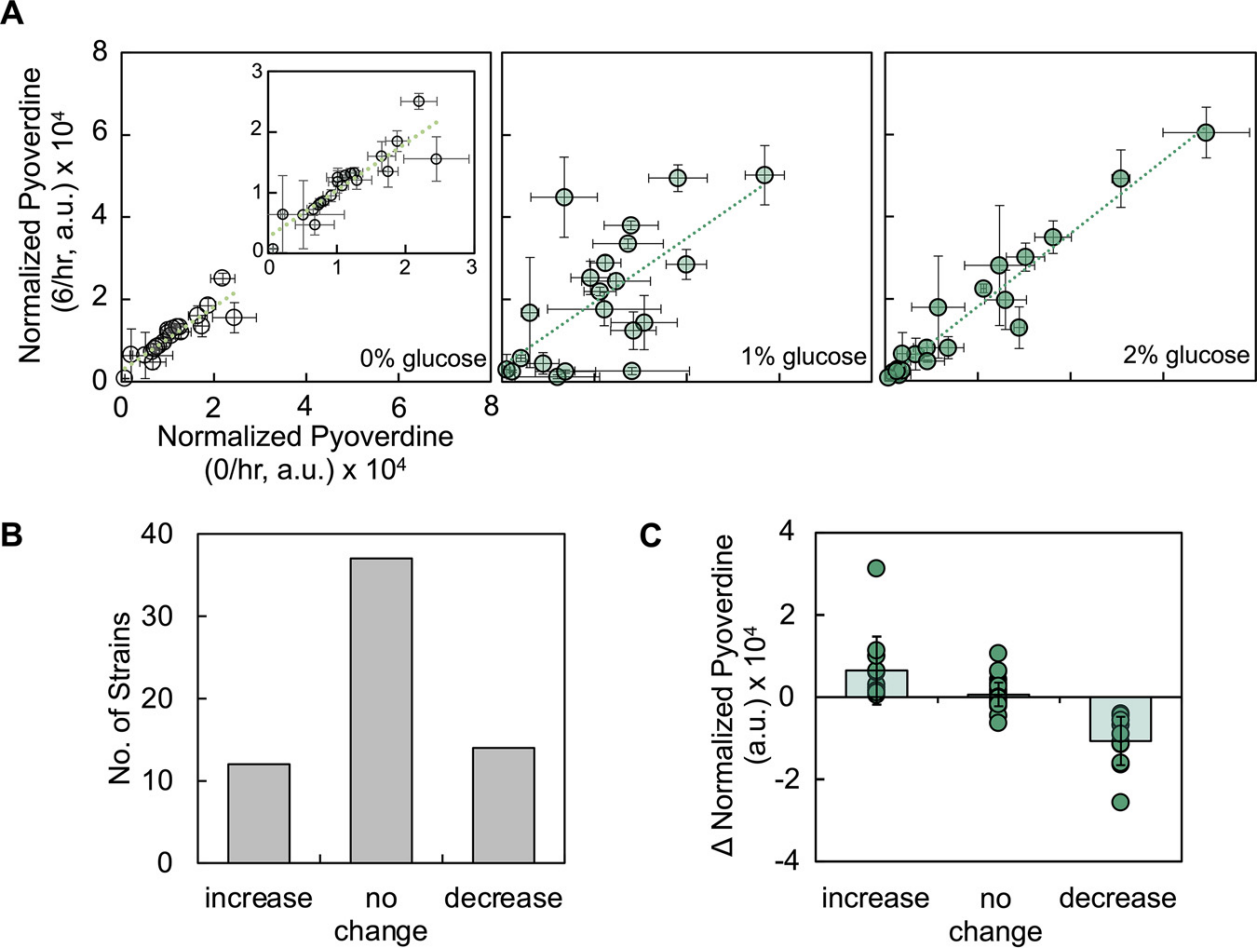
Strains of P. aeruginosa vary in their susceptibility to periodic disturbance at 6/h. (A) Amount of normalized pyoverdine produced by 21 different strains of P. aeruginosa in the undisturbed (0/h) and 6/h conditions when the growth media contained 0% (left, inset – reduced axes), 1% (center), and 2% (right) glucose. The amount of pyoverdine was normalized based on the cell density (OD_600_). Standard deviations from a minimum of six biological replicates were determined. The line drawn serves as a guide; deviations from the line indicate differences in pyoverdine between the undisturbed and disturbed conditions. Raw data are provided in Fig. S5. (B) Number of strains that had an increase, no change, or a decrease in the amount of normalized pyoverdine produced at 6/h relative to 0/h. Significance was assessed using a two-tailed *t* test with a Benjamini-Hochberg correction to protect against false positives. (C) Change in normalized pyoverdine grouped by strains that had an increase, no change, or a decrease at 6/h compared to 0/h. *P < *0.0001 using a Wilcoxon/Kruskal-Wallis test (*P < *0.0001, Shapiro-Wilk test).

We grew biofilms of these strains in medium with 0, 1, and 2% glucose and disturbed each biofilm for 24 h at 6/h, whereupon we quantified the amount of pyoverdine. Our use of different growth media allowed us to study a large parameter space as including different percentages of glucose altered growth rate, biofilm density, and pyoverdine synthesis nonintuitively. For example, while increasing glucose decreased the growth rate for some strains, other strains showed an increase in growth rate, others had a biphasic relationship between growth rate and percentage of glucose in the medium (see Fig. S3). After 24 h of disturbance, we observed that strains showed an increase, decrease, or a lack of change in amount of pyoverdine produced at 6/h relative to 0/h as determined using a two-tailed *t* test (Fig. 4A). Thirty-seven strains/conditions tested did not result in a significant change in the amount of pyoverdine at 6/h (Fig. 4B), whereas 26 strains/conditions showed a significant difference in pyoverdine; 12 strains/conditions showed an increase, and 14 strains/conditions showed a decrease.

10.1128/mSystems.00961-21.3 FIG S3 Growth rate, initial biofilm density, and biofilm density in the 0/h condition cannot account for the change in pyoverdine (or lack thereof) owing to disturbance. (A, top) Growth rates of P. aeruginosa strains grown in medium with 0, 1, and 2% glucose. In all panels, standard deviations from six biological replicates were determined. (A, bottom) Average growth rate grouped by strains that had no change, an increase, or a decrease as a result of periodic disturbance at 6/h (compared to the undisturbed control). Average growth rates were pooled across growth media. A significant difference was not observed when all three conditions were compared (*P = *0.505, one-way ANOVA, *P = *0.174, Shapiro-Wilk). (B, top) Initial biofilm density (after 24 h of growth, no disturbance) of strains grown in the 0, 1, and 2% glucose conditions and separated based on the effect of disturbance. (B, bottom) There was no significant difference in initial biofilm density when strains were grouped based on their response to disturbance. *P = *0.391 (Wilcoxon/Kruskal-Wallis) and *P = *0.0189 (Shapiro-Wilk). (C, top) Biofilm density of P. aeruginosa strains grown in medium with 0, 1, and 2% glucose in the 0/h condition. (C, bottom) There was no significant difference in biofilm density (0/h condition) when strains were grouped based on their response to disturbance. *P = *0.1268 (Wilcoxon/Kruskal-Wallis) and *P < *0.0001 (Shapiro-Wilk). (D, top) Dispersal rate of P. aeruginosa strains grown in medium with 0, 1, and 2% glucose. (D, bottom) There was no significant difference in dispersal rate when strains were grouped based on their response to disturbance. Averages were plotted from three replicates (*P = *0.7747 [Wilcoxon/Kruskal-Wallis] and *P < *0.0001 [Shapiro-Wilk]). Download FIG S3, JPG file, 0.7 MB. Copyright © 2021 Quinn et al. 2021 Quinn et al. https://creativecommons.org/licenses/by/4.0/ This content is distributed under the terms of the Creative Commons Attribution 4.0 International license.

10.1128/mSystems.00961-21.5 FIG S5 Raw data for Fig. 4A. (A) Average amount of normalized pyoverdine produced by P. aeruginosa strains in the 0/h and 6/h conditions with 0% glucose in the growth medium. For all panels, pyoverdine was normalized by cell density (OD_600_), and standard deviations from six biological replicates were determined. *, Statistically significant difference between the 0/h and 6/h conditions (*P ≤ *0.0498, two-tailed t test). Significant changes were determined using a two tailed *t* test with a Benjamini-Hochberg correction to protect against false positives. (B) Average amount of normalized pyoverdine produced by P. aeruginosa strains in the 0/h and 6/h conditions with 1% glucose in the growth medium. (C) Average amount of normalized pyoverdine produced by P. aeruginosa strains in the 0/h and 6/h conditions with 2% glucose in the growth medium. Download FIG S5, JPG file, 0.5 MB. Copyright © 2021 Quinn et al. 2021 Quinn et al. https://creativecommons.org/licenses/by/4.0/ This content is distributed under the terms of the Creative Commons Attribution 4.0 International license.

**FIG 5:**
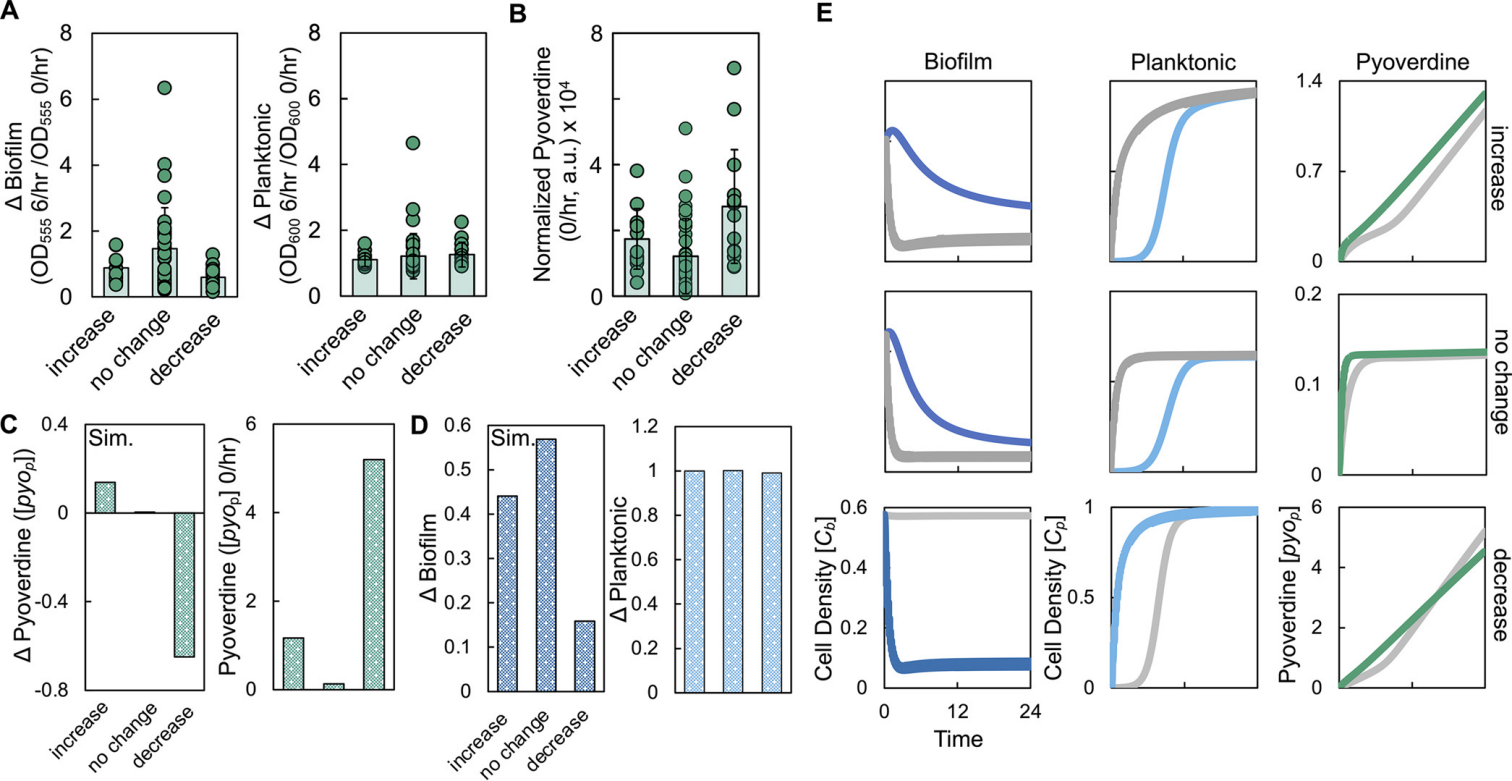
Changes in the density of the biofilm can account for the increase, decrease, or lack of change in the amount of pyoverdine as a result of periodic disturbance. (A) Experimental Δbiofilm (left panel, *P = *0.017, Kruskal-Wallis) and Δplanktonic (right panel, *P = *0.2187, Kruskal-Wallis, Shapiro-Wilk, *P < *0.0001) values due to periodic disturbance at 6/h. Standard deviations from six biological replicates were determined. (B) Amounts of normalized pyoverdine produced in the 0/h condition grouped by strains that had an increase, no change, or a decrease at 6/h compared to 0/h (*P = *0.0011, Kruskal-Wallis, Shapiro-Wilk, *P < *0.0001). (C, left) Simulations showing the difference in pyoverdine between 0/h and 6/h for different pyoverdine synthesis rates. (C, right) Simulations showing the final amount of pyoverdine in the 0/h condition for different pyoverdine synthesis rates. For panels C to E, we used equations 5 to 8. Parameter values, including initial biofilm densities and pyoverdine synthesis rates, are provided in Table S3. *t *=* *24 h. General trends in panels C to E can be found over longer simulation times (see Fig. S6B) and over a range of parameter values (see Fig. S7). For all panels, Sim. = simulation results. (D) Simulated Δbiofilm (left) and Δplanktonic (right) values. (E) Simulations showing the temporal changes in the density of bacteria in the biofilm (left) and planktonic states (center), as well as the amount of pyoverdine (right), for each pyoverdine synthesis rate.

To determine why a disturbance at 6/h affected the amount of pyoverdine synthesized for some strains, but not others, we grouped the strains/conditions as follows based on their responses to disturbance: increase, decrease, and no change. The change in pyoverdine was significantly different across these groupings thus providing support for our grouping approach (Fig. 4C). To determine the impact of periodic disturbance on the distribution of bacteria, we quantified Δbiofilm and Δplanktonic. We observed a significant difference in Δbiofilm as a result of disturbance; Δbiofilm was smallest when a decrease in pyoverdine was observed and greatest when pyoverdine remained unchanged (Fig. 5A). A significant difference was not observed in Δplanktonic (Fig. 5A). Within each group, we quantified initial biofilm density, pyoverdine synthesis (0/h), final biofilm density (0/h), and growth rate. We found that there was a significant difference in pyoverdine synthesis between each group; pyoverdine synthesis was highest in the group that showed a decrease in the amount of pyoverdine, and it was the lowest in the group that did not show a change in pyoverdine amount (Fig. 5B). We did not find any significant differences in growth rate, initial biofilm density, motility, and final biofilm density at 0/h when strains were grouped based on their response to disturbance at 6/h (see Fig. S3). We also did not find a significant relationship between the combined effect of initial biofilm density and pyoverdine synthesis (0/h) on the change in pyoverdine owing to disturbance (*P = *0.1078, multiple linear regression model, least-squares analysis). Finally, we did not find a consistent relationship between the effect of disturbance and the genetic diversity of strains or sequence diversity across proteins involved in pyoverdine synthesis (see Fig. S4). Overall, our analysis identified two key factors that were connected with a strain’s response to disturbance at 6/h: differences in Δbiofilm and pyoverdine synthesis.

10.1128/mSystems.00961-21.4 FIG S4 Phylogenetic analysis of gene and protein sequences of core and pyoverdine synthesis genes and their relationship to the effect of disturbance at 6/h on changes in pyoverdine amount. (A) Phylogenetic relationships between 16 P. aeruginosa strains used in this study using 16S rRNA (left) and 49 concatenated single copy core genes (1) (right). For all panels, circles next to each strain indicate effect of disturbance at 6/h (relative to 0/h) for each percentage of glucose (green = increase, black = no change, red = decrease). (B) Phylogenetic relationships between select genes in pyoverdine synthesis (*pvdT*, *pvdS*, *pvdL*, *pvdE*, and *pvdP*) detectable in 15 strains used in this study. No trends were observed when comparing phylogenetic relationships and the change in pyoverdine owing to disturbance. (C) Concatenated tree with Pvd protein sequences (PvdS, PvdH, PvdF, PvdO, PvdN, PvdM, FpvR, FpvI, PvdA, and PvdQ). No trends were observed when comparing phylogenetic relationships and the change in pyoverdine owing to disturbance. Download FIG S4, JPG file, 0.4 MB. Copyright © 2021 Quinn et al. 2021 Quinn et al. https://creativecommons.org/licenses/by/4.0/ This content is distributed under the terms of the Creative Commons Attribution 4.0 International license.

10.1128/mSystems.00961-21.6 FIG S6 The change in biofilm density between 24 and 48 h of growth. (A) Change in biofilm density. The 24-h value represents the initial density; the 48-h value represents the final density in the 0/h condition. *P = *0.0745, Mann-Whitney (Shapiro-Wilk < 0.0001). Standard deviations from a minimum of three biological replicates for each strain were determined; the OD_555_ was then averaged across these values. (Right panel) Change in biofilm density separated based on the effect of disturbance. Solid bars = 24 h, stripped bars = 48 h. (B) Longer simulation times continue to produce qualitatively similar simulations compared to shorter simulation times that coincide with the time over which our experiments occurred (*t *=* *24 h). Parameters are provided in Table S3. For all panels, Sim. = simulation results. (C) Simulations performed with the same initial biofilm density (*C_b_* = 0.577). These simulation results are qualitatively consistent with the experimental results presented in Fig. 4 and 5, indicating that the assumption that the initial biofilm density changes (as is observed experimentally in panel A of this figure) between strains that show an increase, no change, or a decrease in pyoverdine amount as a result of periodic disturbance can be relaxed. *t *= 24 h. Parameters used for simulation are provided in Table S3. Download FIG S6, JPG file, 0.5 MB. Copyright © 2021 Quinn et al. 2021 Quinn et al. https://creativecommons.org/licenses/by/4.0/ This content is distributed under the terms of the Creative Commons Attribution 4.0 International license.

10.1128/mSystems.00961-21.7 FIG S7 Sensitivity analysis of the parameters in our mathematical model. Changing the values of parameters in our model (equations 5 to 8) leads to qualitatively consistent results compared to our base parameter set (data points with bold outlines; Table S3). In some instances, changes to the simulation outcome were sufficiently small that lines on the plots overlap. For growth and synthesis rates, the *x* axis shows the rate associated with bacteria in the biofilm state; the rate for bacteria in the planktonic state is 20% greater in all cases. *t *= 24 h. Sim. = simulation results. Download FIG S7, JPG file, 0.5 MB. Copyright © 2021 Quinn et al. 2021 Quinn et al. https://creativecommons.org/licenses/by/4.0/ This content is distributed under the terms of the Creative Commons Attribution 4.0 International license.

Next, we used our model to understand why disturbance at 6/h affected pyoverdine synthesis in some, but not all, strains. For these simulations, we varied the pyoverdine synthesis rates (Fig. 5B) and the initial biofilm density (see Fig. S3) in accordance with our experimental results. With respect to the latter, our experimental results show that, on average, initial biofilm density is greatest in the decrease group and lowest in the increase group. Relaxing this assumption by making the initial biofilm density consistent across groupings does not change the qualitative features of our modeling predictions (see Fig. S6). Our simulations predict the general findings of our experiments (Fig. 5C and D). At a high pyoverdine synthesis rate, the total amount of pyoverdine produced is reduced at 6/h (compared to 0/h; Fig. 5C). This coincided with a small value of Δbiofilm and a value of Δplanktonic that was ∼1 (Fig. 5D). A further reduction in pyoverdine synthesis rate served to increase the amount of pyoverdine produced in the 6/h condition (Fig. 5C). This coincided with an intermediate value of Δbiofilm and a value of Δplanktonic that was ∼1 (Fig. 5D). Finally, a further reduction in pyoverdine synthesis rate resulted in a very small increase in the amount of pyoverdine produced at 6/h (Fig. 5C). This coincided with a large value of Δbiofilm and a value of Δplanktonic was ∼1 (Fig. 5D). Overall, our simulations capture the trends of our experimental findings.

To understand the mechanism behind these findings, we simulated temporal changes in the density of bacteria in the biofilm and planktonic states, and the amount of pyoverdine under the 0/h and 6/h conditions. Our simulations predict that, in general, the density of bacteria in the biofilm state was reduced at 0/h owing to natural dispersal, which was consistent with our experimental findings (see Fig. S6). When pyoverdine synthesis is high and at 0/h, the amount of pyoverdine produced is high owing to a high density of bacteria in both the biofilm and planktonic states. Disturbance at 6/h reduces the density of the biofilm by transitioning bacteria into the planktonic state at a rate greater than natural dispersal. Over 24 h, this does not result in a significant increase in the final density of bacteria in the planktonic state likely owing to the carrying capacity of the medium (Fig. 5D and E). While these bacteria have increased pyoverdine synthesis and growth owing to their position in the planktonic state, the reduction in pyoverdine synthesized by, and diffused from, bacteria in the reduced biofilm state cannot be compensated for by bacteria in the planktonic state. Thus, the overall amount of pyoverdine is reduced.

At an intermediate pyoverdine synthesis rate, the amount of pyoverdine produced by bacteria in the biofilm at 0/h is reduced owing to reductions in both pyoverdine synthesis rate and biofilm density. Disturbance at 6/h transitions bacteria from the biofilm into the planktonic state more rapidly than natural dispersal processes. This early and rapid increase in bacteria in the planktonic state serves to increase the total amount of pyoverdine synthesized as bacteria in this state as they take advantage of the higher pyoverdine synthesis and growth rates. When combined with the pyoverdine diffused from bacteria in the biofilm state, the overall amount of pyoverdine is increased.

Finally, at a low pyoverdine synthesis rate, the amount of pyoverdine produced by bacteria in the biofilm at 0/h is further reduced. As above, disturbance at 6/h transitions the bacteria into the planktonic state more quickly compared to natural dispersal. Although these bacteria produce pyoverdine at a higher synthesis rate, it is insufficient to lead to an appreciable increase in the total amount of pyoverdine synthesized. Thus, the total amount of pyoverdine synthesized remains largely unchanged between the undisturbed and 6/h conditions. These predictions are consistent when the values of parameters in our model, including the amount of pyoverdine and bacteria transitioned from the biofilm to the planktonic state owing to disturbance, are varied (see Fig. S7). Overall, interactions between pyoverdine synthesis rate and the relative change in biofilm density determines the effect that periodic disturbance has on the amount of pyoverdine produced by P. aeruginosa strains at 6/h.

## DISCUSSION

We have shown that periodically disturbing the spatial structure of a biofilm of P. aeruginosa using a physical force can affect pyoverdine production. Our model suggests that changes in the amount of pyoverdine, or lack thereof, can be attributed to the change in the density of bacteria in the biofilm state owing to disturbance and the synthesis rate of pyoverdine. While we cannot rule out that additional factors may contribute to any changes in pyoverdine production, such as differences in oxygenation attributed to shaking, we did not find significant associations between initial biofilm density, biofilm density at 0/h, growth rate, diversity across the pyoverdine locus, or motility. It is likely that there is heterogeneity in pyoverdine expression in our system (35). However, this is unlikely to confound our results or mechanism as our model, which predicts the qualitative trends in our data over a wide parameter space (see Fig. S1 and S7), considers average population behavior only. Our analysis focused on a disturbance frequency of 6/h, which was driven by our initial observation that this frequency resulted in the greatest significant change in the quantity of pyoverdine produced from PA14 (Fig. 2). However, additional shaking frequencies, such as those greater or less than 6/h, may alter pyoverdine production differently for each strain. Moreover, the type of force applied to the biofilm may differentially affect pyoverdine production. While we used linear shaking to test the generality of fluctuations in physical force and their effect on the production of virulence factors, other types of physical forces will have additional considerations. For example, changes in flow rate would serve to remove secreted virulence factors from the system entirely, which may serve to further alter production. Thus, our experimental findings and model may not be relevant in all situations where periodic disturbances that affect spatial structure occur. Finally, while the molecular players involved in quorum sensing, such as the Pseudomonas quinolone signal (51), can influence pyoverdine production, production of pyoverdine is largely regulated through non-quorum sensing means (31). Thus, although we cannot exclude the possibility that quorum sensing is influencing our findings, it likely plays a secondary role, which certainly warrants future exploration.

Previous work has noted differences in virulence factor expression, including pyoverdine, from strains isolated from the clinic and environment. Strains isolated from infections of various durations (52) and patients (53) show diversity in virulence factor expression. Differences in growth environments can lead to diverse selective pressures (54). Pressures unique to each strain might have shaped their ability to resist, or be altered by, periodic disturbance in terms of the amount of pyoverdine produced. For example, those strains where a reduction in pyoverdine was observed might have evolved in areas that are less prone to fluctuations in physical forces. Thus, they are ill adapted to such conditions, which results in a decrease in pyoverdine. Conversely, strains where changes in pyoverdine amount were not found might have evolved in areas that face frequent changes in physical force. Adapting to this type of environment would allow the strain to maintain a relatively constant amount of pyoverdine, which could facilitate the maintenance of virulence. While this is plausible, we did not find significant associations between isolation source (e.g., wound, urine, blood, etc.) and the effect of disturbance (not shown).

While our manuscript focused on the impact of disturbance to spatial structure, we also characterized the relationship between pyoverdine production and biofilm density across strains of P. aeruginosa. Previous work has found that biofilm formation can promote the production of pyoverdine but that this was not consistent for all strains (34). While our initial observation that increasing glucose concentration increased pyoverdine synthesis in strain PA14 was consistent with previous work performed in Pseudomonas sp. (48), this observation was not consistent for all strains (see Fig. S5). The relationship between pyoverdine synthesis and carbon source across strains is complex and may reflect a combination of carbon source preference (55), evolutionary constraints (50), and strain-specific genetic differences (56). In contrast to previous work (49), we did not observe that increasing the percentage of glucose in the medium increased biofilm density. However, this can likely be attributed to differences in methodology between our study and the previous study (49) in terms of growth medium (57), temperature (58), and forces under which biofilms were formed (59), all of which have been previously shown to impact biofilm density.

Disruption of virulence factor function (10), including pyoverdine (60, 61), has shown promise toward attenuating pathogenesis. However, these approaches place selective pressure directly on the virulence factor. Changes to spatial structure through the periodic application of physical force might spread the selection pressure out over multiple targets (e.g., genes involved in virulence factor production, biofilm formation, and/or quorum sensing). This could serve to limit evolution against such forces while reducing virulence factor production. Such an approach would require careful considerations; if disturbances increased the amount of virulence factor, this could further the infection process. Counterintuitively, disturbances may make the population more susceptible to antibiotics since there can be a negative correlation between antibiotic resistance and production of certain virulence factors (62). Conversely, if periodic disturbance decreased the amount of virulence factor, this could reduce infection severity and thus provide a novel mechanism to treat infections.

## MATERIALS AND METHODS

### Strains and growth conditions.

P. aeruginosa strains used in this study are listed in Table S2. All experiments were performed in modified King’s A medium (2% peptone [Fisher Scientific, Waltham, MA], 0.5% potassium sulfate, [Acros Organics, Fisher Scientific], 17 mM magnesium chloride [Alfa Aesar, Ward Hill, MA]) with different glucose concentrations (0, 1, or 2% glucose [VWR, Radnor, PA]). Single colonies of P. aeruginosa isolated from Luria-Bertani (LB) agar medium (MP Biomedicals, Solon, OH) were shaken overnight (250 rpm and 37°C) in 3 ml of liquid LB medium in culture tubes (Genesee Scientific, Morrisville, NC). Where applicable, gallium nitrate (Acros Organics) was added to the final concentration indicated. We used the Innovotech (Edmonton, Alberta, Canada) MBEC biofilm inoculator to grow biofilms (37). We grew overnight cultures of P. aeruginosa, washed the cells in fresh King’s A medium, and diluted them 1,000-fold into fresh modified King’s A medium. We then placed 150 μl of culture into the wells of the plate and shook the plate at 110 rpm at 25°C for 24 h.

### Dispersal of cells from biofilm.

We grew biofilms in modified King’s A medium. After 24 h of growth, we removed the plate and washed the pegs in 200 μl of fresh King’s A medium to remove any unadhered cells. The washed biofilms were placed in 200 μl of fresh modified King’s A medium, and the plate was placed in a Victor X4 plate reader preset to 25°C. The biofilms were then shaken once with an amplitude of 0.1 mm (fast setting, frequency = 4,800 mm/min, 10 s per shaking event, linear shaking feature which shakes the plate along the *x* axis). To measure the density of bacteria in the planktonic state, we removed 10 μl from the medium surrounding the peg. To measure the density of bacteria in the biofilm, the peg was removed from the lid of the MBEC plate using sterilized forceps. The peg was placed in 200 μl of fresh LB medium and vortexed the sample to remove bacteria. We counted the CFU in the King’s A medium surrounding the plate by plating a serial dilution on LB agar.

### Biofilm staining.

We followed a previously published protocol (63). We grew biofilms as described above. After 24 h, we washed the pegs (which remained attached to the lid of the microplate) with 200 μl of fresh modified King’s A medium for 10 s. The washed pegs were placed in 125 μl of 0.1% crystal violet (Acros Organics) for 10 min. We then washed the stained pegs in 200 μl of ddH_2_O four times to remove excess crystal violet. Finally, we transferred the pegs to 200 μl of 30% acetic acid (Fisher Scientific) for 10 min to remove crystal violet from the biofilms. The amount of solubilized crystal violet was measured using OD_555_ in a Victor X4 plate reader (Perkin-Elmer, Waltham, MA). To measure biofilm density in the absence of MBEC plate, we followed the procedure outlined an a previous study (49). Single colonies of P. aeruginosa were grown in 3 ml of LB media for 24 h at 37°C and 250 rpm. Bacteria were diluted 200-fold into 200 μl of King’s A media containing 0 and 2% glucose, respectively, in a 96-well microplate. The microplate was incubated at 37°C without shaking. After 24 h, bacteria in the planktonic state were carefully aspirated, and the biofilm adhered to the wells was washed three times with 1× phosphate buffer saline (PBS). Biofilms were stained with 200 μl of 0.25% (wt/vol) crystal violet solution for 15 min at room temperature, washed three times with PBS, and allowed to dry for 20 min. Then, 200 μl of 95% ethanol was added to the wells, followed by incubation at room temperature for 20 min. The absorbance was measured at 555 nm using a Victor X4 plate reader.

### Periodic disturbance experiments.

We grew biofilms in modified King’s A medium. After 24 h, we removed the plate and washed the pegs in 200 μl of fresh King’s A medium to remove any unadhered cells. The washed biofilms were placed in 200 μl of fresh modified King’s A medium, and the plate was placed in a Victor X4 plate reader preset to 25°C. The plate was then periodically shaken (fast setting, frequency = 4,800 mm/min, 10 s per shaking event, linear shaking feature which shakes the plate along the *x* axis, amplitude = 0.1 mm) at the frequency indicated. After 24 h of growth in the plate reader, we removed the lid from the plate and measured the cell density (OD_600_) and the concentration of pyoverdine using the Victor X4 microplate reader. Pyoverdine (arbitrary units) normalized by OD_600_.

### Measuring expression of GFP.

We prepared competent P. aeruginosa PA14 as described previously (64). We transformed these cells with plasmid pAB1 (confers ampicillin resistance [65]), which contains an IPTG (isopropyl-β-d-thiogalactopyranoside)-inducible copy of enhanced GFP (eGFP). We grew biofilms of these GFP-expressing bacteria as described above under “Strains and growth conditions.” After washing the pegs to remove any unadhered cells, we placed the biofilms into fresh modified King’s A medium that contained, or did not contain as a control, 1 mM IPTG (Thermo Fisher). We then shook the biofilms periodically (or not at all as a control, 0/h) as described under “Periodic disturbance experiments*”* above. After 24 h, we quantified OD_600_ and GFP (λ_excite_, 485 nm; λ_emission_, 510 nm) using a Victor X4 microplate reader. GFP was normalized by OD_600_.

### RNA extraction and cDNA synthesis.

To isolate total RNA, the Qiagen RNeasy minikit was used following the RNAprotect bacterial reagent handbook, with modifications. P. aeruginosa biofilms were grown as described above. From the MBEC plate, ∼180 μl of medium was collected from each well and placed into 1.5-ml centrifuge tubes. Collected medium was then centrifuged for 2 min at 12,000 rpm. Pelleted cells were resuspended in 200 μl of RNase free water. Each tube was then centrifuged again for 2 min at 12,000 rpm. The remaining supernatant was discarded. After centrifugation, 30 μl of a 10 mg/ml solution of lysozyme (MP Biomedicals) in 1× Tris-EDTA (Thermo Fisher) was added to each centrifuge tube, followed by incubation at room temperature for 20 min with vortexing every 2 min. The total RNA was then extracted according to the manufacturer’s recommended protocol, including the optional in-column DNase digestion using the RNase-Free DNase set. After RNA extraction, we used a Qiagen DNase Max kit according to the Quick Start protocol provided by the manufacturer. cDNA synthesis was performed using Bio-Rad reverse transcription supermix according to the manufacturer’s recommendations in a Bio-Rad C1000 touch thermal cycler.

### RT-qPCR.

Quantitative real-time PCR was performed using the Bio-Rad iTaq Universal SYBR green Supermix and using a Bio-Rad CFX96 touch real-time PCR detection system. Expression of *rpoD* was detected using the primers *rpoD *F (GGCGAAGAAGGAAATGGTC) and *rpoD* R (CAGGTGGCGTAGGTGGAGAA) (66) (Sigma-Aldrich, Darmstadt, Germany). Expression of *pvdA* was quantified using the primers *pvdA *F (GACTCAGGCAACTGCAAC) and *pvdA* R (TGTCCAGGAACAGCACTTC) (Sigma-Aldrich). The parameters for each RT-qPCR cycle used were as follows: 95°C for 3 min, 95°C for 10 s, 63°C for 30 s, 63°C for 31 s, and 63°C for 5 s. A total of 40 cycles, followed by melting-curve analysis, were performed. Amplicon specificity and size were confirmed using agarose gel electrophoresis. The average threshold cycle (*C_q_*) was normalized using the Δ*C_T_* method (67). All *C_T_* values were normalized using *rpoD*.

### Measuring diffusion of pyoverdine.

Biofilms of strain PA14 were grown for 24 h in modified King’s medium with 0 or 2% glucose at 110 rpm and 25°C. The following day, biofilms were washed in 200 μl of 1× PBS to remove any unadhered bacteria, which limited the amount of pyoverdine produced by bacteria dispersed from the biofilm into the surrounding medium. To further limit any natural dispersal or bacterial growth, biofilms were placed in 1× PBS for 1 h. We then filtered the medium surrounding the biofilm using a 0.45-μm filter (Genesee Scientific) to remove any dispersed bacteria and quantified pyoverdine in the cell-free medium. Pyoverdine values were blanked using background fluorescence produced from 1× PBS.

### Growth rate.

We determined the basal growth rate of the P. aeruginosa strains in the absence of biofilm formation. We grew each strain separately overnight in LB medium. The following day, each strain was diluted 200-fold into 200 μl of fresh modified King’s A medium inside a 96-well plate. The plate was shaken at 250 rpm and at 25°C until the bacteria reached exponential phase (∼4 h). We then measured the OD_600_ at regular intervals. We plotted a linear line through a plot of the OD_600_ versus time in h. The growth rate was determined using the slope of a linear line.

### Dispersal rate.

Single colonies were grown overnight in 3 ml of LB medium. The following day, 1 μl of bacteria was placed in the center of 5 ml of modified King’s medium containing different percentages of glucose in the wells of a 6-well plate. Cultures were incubated for 24 h at 25°C. The radius from the center of the colony was measured using a ruler in three representative locations and was subsequently averaged for each replicate. The growth rate was determined from three biological replicates.

### Phylogenetic analysis.

Currently available draft genome assemblies were downloaded from the National Center for Biotechnology Information. Nucleotide sequences for 16S rRNA were aligned using MUSCLE (68), followed by phylogenetic analysis in MegaX v10.1.8. A maximum-likelihood tree was generated using the general time reversible model after 100 bootstrap iterations. Phylogenetic analysis was also performed using 49 concatenated single copy core genes from all P. aeruginosa draft genomes in KBase (69) using the application “Insert GenomeSet into Species Tree,” which employs FastTree2 (70). To find genes in the synthesis of pyoverdine, open reading frames and annotations were performed with RAST (71). Amino acid sequences for each protein were either concatenated or analyzed alone by aligning with MUSCLE and generating a maximum-likelihood tree using the LG model after 100 bootstraps. Protein sequences for *pvd* genes that were detectable in 15 of 16 of the strains were used for concatenation. Proteins PvdT, PvdS, PvdL, PvdE, and PvdP were analyzed separately because they represented key steps in the pyoverdine process.

### Statistical analysis.

Statistical analysis as indicated in the text or figure legend. Unpaired *t* tests (unequal variance) were performed using Microsoft Excel (Redmond, WA). A Shapiro-Wilk test was used to assess normality. When evaluating the significance of changes in pyoverdine amount for strains grown in different media, we applied a Benjamini-Hochberg correction with a false discovery rate of 0.15. Additional tests were performed in JMP Pro 14 (SAS Institute, Inc., Cary, NC).

### Model development and assumptions.

We used the general modeling framework presented in earlier studies (72, 73) as the basis to develop our model. We used four ordinary differential equations, which included the production and decay of pyoverdine (equations 1 and 3) and growth of bacteria (equations 2 and 4).

(1) $$\frac{d[\mathrm{py}o_{b}]}{\mathrm{dt}}\;=-\beta [\mathrm{py}o_{b}]\;+\;k_{\mathrm{sb}}C_{b}\;-\;k_{d}[\mathrm{py}o_{b}]$$

(2) $$\frac{dC_{b}}{\mathrm{dt}}\;=\;–\gamma C_{b}\;+\;\mu_{b}C_{b}\left(1\;–\;\frac{C}{C_{\max}}\right)\;–\;\frac{\delta }{A\;+\;[\mathrm{py}o_{b}]}C_{b}$$

(3) $$\frac{d[\mathrm{py}o_{p}]}{\mathrm{dt}}\;=\;–\beta [\mathrm{py}o_{b}]\;+\;k_{\mathrm{sp}}C_{p}\;–\;k_{d}[\mathrm{py}o_{p}]$$

(4) $$\frac{dC_{p}}{\mathrm{dt}}\;=\;\gamma C_{b}\;+\;\mu_{p}C_{p}\left(1\;–\;\frac{C}{C_{\max}}\right)\;–\;\frac{\delta }{A\;+\;[\mathrm{py}o_{b}]}C_{p}$$

In these equations and in equations 5 to 8 below, subscripts *b* and *p* represent parameters that are associated with bacteria in the biofilm and planktonic states, respectively. *C* represents cell density, *C*_max_ represents the carrying capacity, *k_s_* (μM/h) represents the synthesis rate constant of pyoverdine, “[*pyo*]” (μM) represents the concentration of pyoverdine, *k_d_* (/h) represents the degradation rate of pyoverdine, μ represents growth rate (/h), γ represents the fraction of bacteria moving from the biofilm to the planktonic state owing to natural processes, β represents the fraction of pyoverdine that diffuses from the biofilm to the planktonic state, δ (μM/h) represents the maximal rate at which growth is reduced owing to lack of iron uptake via pyoverdine, *A* (μM) represents the concentration of pyoverdine that leads to half maximal growth reduction, α is the frequency at which an amount of bacteria and pyoverdine, ε is transferred from the biofilm state into the planktonic state, σ is the fraction of bacteria that are recruited to the biofilm, and *t* represents time (h). Simulations were performed in MATLAB (R2020b; MathWorks, Inc., Natick, MA) using ode23s (*t *=* *24 h). Additional parameters may be found in Table S3 in the supplemental material.

To develop our model, we consider two populations of bacteria: one population in the biofilm state (*C_b_*) and one population in the planktonic state (*C_p_*). Both populations grow according to logistic growth at a growth rate of μ_b_ and μ_p_, respectively. Each population has its own carrying capacity, *C*_max_, which is normalized to 1. Thus, total cell density is always scaled to a maximum of 1. Populations in the biofilm and planktonic states produce pyoverdine according to first-order kinetics at a rate of *k_sb_* and *k_sp_*, respectively. Pyoverdine production is dependent upon the density of bacteria in each state. For simplicity, we assume that pyoverdine degrades according to first-order kinetics at a rate of *k_d_*, the value of which is consistent for both populations (74). Pyoverdine produced by the bacteria in the biofilm state diffuses into the surrounding medium at a rate of β. Bacteria from the biofilm state can move to the planktonic state at a rate of γ to account for natural dispersal, diffusion, and sloughing (75).

Consistent with previous studies that have examined the biosynthesis and utilization of pyoverdine (76, 77), we modeled the impact of pyoverdine on cell growth as a modified Michaelis-Menten type equation. δ represents the maximal rate at which growth is reduced owing to lack of iron uptake via pyoverdine, while *A* represents the concentration of pyoverdine that leads to half-maximal growth. The modified Michaelis-Menten term in our equations accounts for the observation that reducing pyoverdine functionality (44) or by removing the ability of P. aeruginosa to synthesize pyoverdine via gene deletion (78) reduces growth and biomass accumulation. As pyoverdine accumulates in the medium, any trace iron will be sequestered, which will facilitate growth. Thus, in the extreme case where pyoverdine is minimally produced, or not produced at all, growth is reduced. This is consistent with our data and previous literature (44, 79) showing that inhibiting pyoverdine functionality through deletion (see Fig. S1B) or the introduction of gallium (Fig. 2H) significantly reduces growth. However, as the concentration of pyoverdine reaches its maximum in the growth environment, growth will occur at the fastest possible rate (as dictated by μ in the logistic term). We note that while a sufficiently high concentration of iron can attenuate pyoverdine production (31), we did not explicitly model this interaction as we grew the bacteria in an iron limited environment and thus this could not be reached in our system.

### Simulating the effect of periodic disturbance.

To simulate the effect of periodic disturbance, we take a similar approach to previous work (72). At a given frequency (α), an amount of bacteria and pyoverdine (ε) is transferred from the biofilm state into the planktonic state. Thus, we modify equations 1 to 4 as follows:

(5) $$\frac{d[\mathrm{py}o_{b}]}{\mathrm{dt}}\;=-\varepsilon [\mathrm{py}o_{b}]\alpha (t_{1})\;–\;\beta [\mathrm{py}o_{b}]\;+\;k_{\mathrm{sb}}C_{b}\;-\;k_{d}[\mathrm{py}o_{b}]$$

(6) $$\frac{dC_{b}}{\mathrm{dt}}\;=\;\sigma C_{p}\;–\;\varepsilon C_{b}\alpha (t_{1})\;–\;\gamma C_{b}\;+\;\mu_{b}C_{b}\;+\;\mu_{b}C_{b}\left(1\;–\;\frac{C}{C_{\max}}\right)\;–\;\frac{\delta }{A\;+\;[\mathrm{py}o_{b}]}C_{b}$$

(7) $$\frac{d[\mathrm{py}o_{p}]}{\mathrm{dt}}\;=\;\varepsilon [\mathrm{py}o_{b}]\alpha (t_{1})\;+\;\beta [\mathrm{py}o_{b}]\;+\;k_{\mathrm{sp}}C_{p}\;–\;k_{d}[\mathrm{py}o_{p}]$$

(8) $$\frac{dC_{p}}{\mathrm{dt}}\;=\;–\sigma C_{p}\;+\;\varepsilon C_{b}\alpha (t_{1})\;+\;\gamma C_{b}\;+\;\mu_{p}C_{p}\left(1\;–\;\frac{C}{C_{\max}}\right)\;–\;\frac{\delta }{A\;+\;[\mathrm{py}o_{b}]}C_{p}$$

where *α*(*t*_1_) is a dirac delta function where *t*_1_ = *kT*_0_, and *T*_0_ represents the frequency of transfer events. ε accounts for the movement of both bacteria and pyoverdine so that any pyoverdine that is sequestered by the bacteria (e.g., through cell-cell contacts in the biofilm) at the moment of movement owing to periodic disturbance is transferred into the planktonic state, and σ represents the shaking dependent transfer of bacteria from the planktonic state back to the biofilm state. We included the σ term to account for previous studies that have shown that increased shear force causes recruitment of bacteria from the planktonic state back to the biofilm state (42, 43).

### Parameter estimation.

Parameters used in our mathematical model can be found in Table S3. We estimated the maximum growth rate of bacteria in the planktonic state (*μ_p_*) from previously published work (80). Previous studies have demonstrated that spatial organization (81, 82), including biofilms (45, 83), can reduce access to nutrients to cells on the inside of spatial structures, including biofilms. In contrast, bacteria in the planktonic state have greater access to nutrients as they will not be surrounded by as many competitors compared to bacteria in the biofilm state. Accordingly, we assume that the growth rate of bacteria in the biofilm is less than the growth rate of bacteria in the planktonic state. Thus, we estimated that *μ_b_* is reduced by 20% relative to *μ_p_* to account for nutrient limitation in the biofilm (73).

Previous work has indicated that the average amount of pyoverdine synthesized by a colony of P. aeruginosa PAO1 is ∼2 μM after 7 h of growth (35). Since our experiments occur over 24 h, we estimated that the total amount of pyoverdine synthesized would be approximately 5 to 8 μM. We therefore scaled the pyoverdine synthesis rate, *k_sb_*, such that the final amount of pyoverdine produced by a population (both *C_b_* and *C_p_* combined) of strain PA14 was within this range of pyoverdine concentration. Note that our model and experimental measurements consider pyoverdine synthesis performed by a mature biofilm, and not pyoverdine production during the formation of a biofilm from a population initiated from the planktonic state.

Previous work has indicated that the rate of *pvdS* synthesis is increased ∼5-fold in planktonic cells compared to cells in the biofilm (13). Increases in proteins involved in pyoverdine synthesis are also observed in planktonic bacteria (84). Moreover, aggregates from planktonic bacteria show increased pyoverdine amounts (2-fold) and *pvdS* expression (4-fold). However, other papers have indicated that biofilm formation is required for pyoverdine production (85). To account for these conflicting observations, we took an intermediate approach and adjusted the value of *k_sp_* such that it is ∼20% greater than *k_sb_*. Relaxing this assumption, such that *k_sp_ = k_sb_* does not qualitatively affect our model predictions (see Fig. S1F).

The degradation rate of pyoverdine (*k_d_*) was estimated using a previously published value that examined the dilution of pyoverdine (0.0085/min or ∼0.5/h) in growing cells (86). While passive degradation of pyoverdine is likely to occur in our system, most pyoverdine appears to be recycled (87). To account for the stability of pyoverdine, we reduced the order of magnitude of the degradation rate by 100-fold (∼0.005/h).

To estimate the diffusion rate of pyoverdine, β, we considered that the vast majority, but not all, pyoverdine will immediately diffuse away from its production source. We assumed that not all pyoverdine would diffuse away from the biofilm as previous work has indicated that cell-cell contacts can augment the accumulation of pyoverdine, likely by limiting its diffusion (35). Thus, we estimated that 0.7 to 0.9 of pyoverdine from the biofilm will diffuse into the surrounding medium that contains bacteria in the planktonic state. We note that because of our experimental setup (grow biofilm for 24 h, wash in fresh medium, place biofilms in fresh medium, and begin shaking in the microplate reader), diffusion of pyoverdine from the biofilm to the planktonic state is the initial and predominant direction in which pyoverdine moves in our system.

Dispersal from a biofilm may be due to passive diffusion, sloughing, or active dispersal mechanisms (75). To estimate γ, we considered previously published data that showed that 1 to 10% of Pseudomonas disperse from mature biofilms (growth for 4 days) (88, 89) and occurs in response to nutrient stress/deprivation (90). However, because our biofilms are substantially younger (24 h) and we supply fresh medium just prior to shaking, we estimate that less than 1 to 10% of the population (when scaled to *C_m_*, this would represent *C *=* *0.01 to 0.1) will undergo dispersal over the 24-h period during which the plate is periodically perturbed. Finally, because we chose to grow the bacteria in an iron limited environment, iron driven biofilm dispersal likely does not occur. Indeed, such dispersal appears to commence occurring if 50 μM ferric sulfate is provided, which is substantially higher than any trace amount of iron or iron-complexed molecule that would be included in our system (91). Accordingly, we estimated that the dispersal rate of bacteria from the biofilm (*C_b_*) to the planktonic state (*C_p_*) will be approximately 0.001 to 0.0001/h (where *C*_max_ for *C_b_* and *C_p_* = 1). The values of δ and *A* were fit such that the total amount of pyoverdine produced by a population ([*pyo_b_*]+[*pyo_p_*]) of strain PA14 without periodic disturbance was approximately 5 to 6 μM over the total simulated time (24 h) (35).

ε was estimated using data presented in Fig. 1B showing the effect of a single shaking event on the number of bacteria dispersed from the biofilm (0.23 average decrease in biofilm density and 0.26 increase in planktonic density). Thus, we estimated that ε would lie within the approximate range of 0.1 to 0.3. σ was fit to a third order polynomial function such that the value of σ increased with increasing shaking frequency. We fit this value to a polynomial function (with the equation σ = (3.95 × 10^−4^)*x*^3^ −(7.83 × 10^−3^)*x*^2^ + (5.39 × 10^−2^)*x* − 4.84 × 10^−4^, norm of residuals = 0.0011277) since previous work has shown a nonlinear increase in biofilm density with increasing shear force (42). We note, however, if the shear force is too high, biofilm density decreases. We do not appear to achieve this sufficiently high shear force in our system because at the highest shaking frequency tested (30/h), biofilm density is not significantly reduced compared to 0/h (Fig. 1C, Fig. 2C and Fig. 3D). Finally, α, which represents the frequency of transfer events, was matched to the experimental condition (6 shakes/h = transfer frequency of 1/6).

In accordance with our experimental protocol where a biofilm is formed prior to shaking, all simulations were initialized with 0.133 μM pyoverdine in the biofilm state and a population of bacteria in the biofilm state where *C_b_* = 0.577 (where *C*_max_* *=* *1). Note that in our system, *C_b_* = *C*_max_ only when dispersal of bacteria due to natural processes does not occur. We used values of 0.133 μM and *C_b_* = 0.577 since these values allowed a near-constant biofilm density to be maintained in the absence of shaking (Fig. 2F). However, as dictated by trends in our experimental data for strains other than PA14, the initial biofilm density was varied (see Table S3).
