# Supplementary material for: Periodically Disturbing the Spatial Structure of Biofilms Can Affect the Production of an Essential Virulence Factor in Pseudomonas aeruginosa
Source: mSystems. 2021 Sep 28;6(5):e00961-21. doi: 10.1128/mSystems.00961-21 (PMC8547473; doi:10.1128/mSystems.00961-21)
Supplement: TABLE S1 [file msystems.00961-21-st001.docx]

**Supplemental Table S1.**

| **Pathogen** | **Virulence Factor** | **Reference** |
| --- | --- | --- |
| **High amount of spatial structure increases expression of virulence factor** | | |
| *Staphylococcus aureus* | Exotoxin proteins are highly expressed in biofilms owing to the accumulation of AIP. | (2) |
| *Enterococcus* *faecalis* | Gelatinase and serine protease expression is increased in mature biofilms. | (3) |
| *Burkholderia cepacia* | Virulence factor production and swarm motility is highly expressed in biofilm the state | (4, 5) |
| *P. aeruginosa* | Rhamnolipid production increases in biofilms. | (6) |
| *Vibrio* *parahaemolyticus* | Increased expression of exoproteases is observed in biofilms. | (7, 8) |
| **Low amount of spatial structure increases expression of virulence factor** | | |
| *S. aureus* | Surface attachment proteins are highly expressed in bacteria that have dispersed from the biofilm owing to a reduction in AIP. | (2) |
| *P. aeruginosa* | Pyoverdine expression is increased in bacteria dispersed from the biofilm. | (9, 10) |
| *V. cholerae* | Surface attachment proteins and toxins are expressed in bacteria that have dispersed from the biofilm. | (11, 12) |
| *Clostridium perfringens* | Expression of several virulence factors is increased when cells are in the planktonic state. | (13) |
| *Gardnerella vaginalis* | Expression of vaginolysin toxin is increased when bacteria are in the planktonic state. | (14) |
