# Supplementary figures and images for: Periodically Disturbing the Spatial Structure of Biofilms Can Affect the Production of an Essential Virulence Factor in Pseudomonas aeruginosa
Source: mSystems. 2021 Sep 28;6(5):e00961-21. doi: 10.1128/mSystems.00961-21 (PMC8547473; doi:10.1128/mSystems.00961-21)

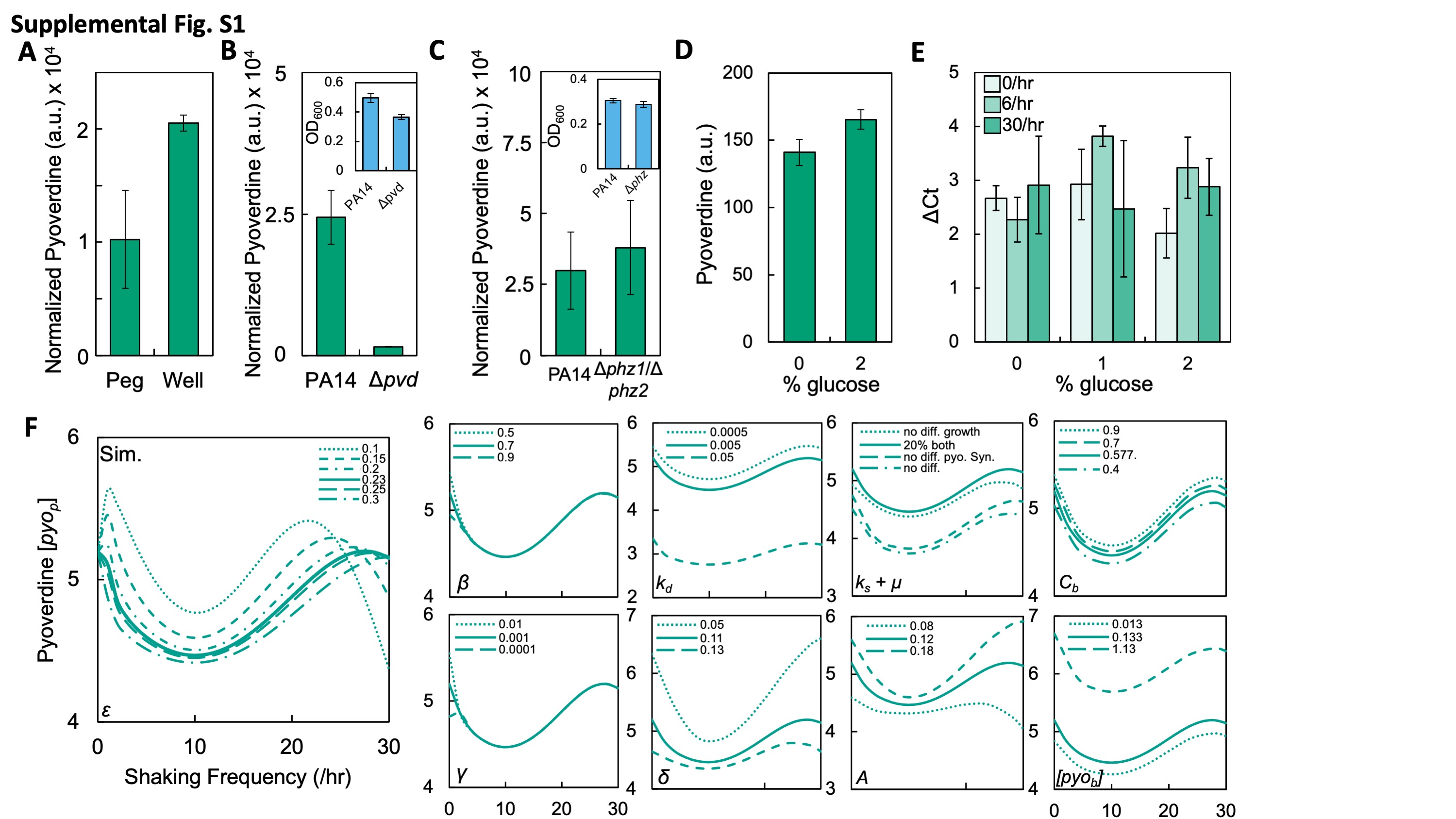

Supplement: FIG S1 [file msystems.00961-21-sf001.jpg]

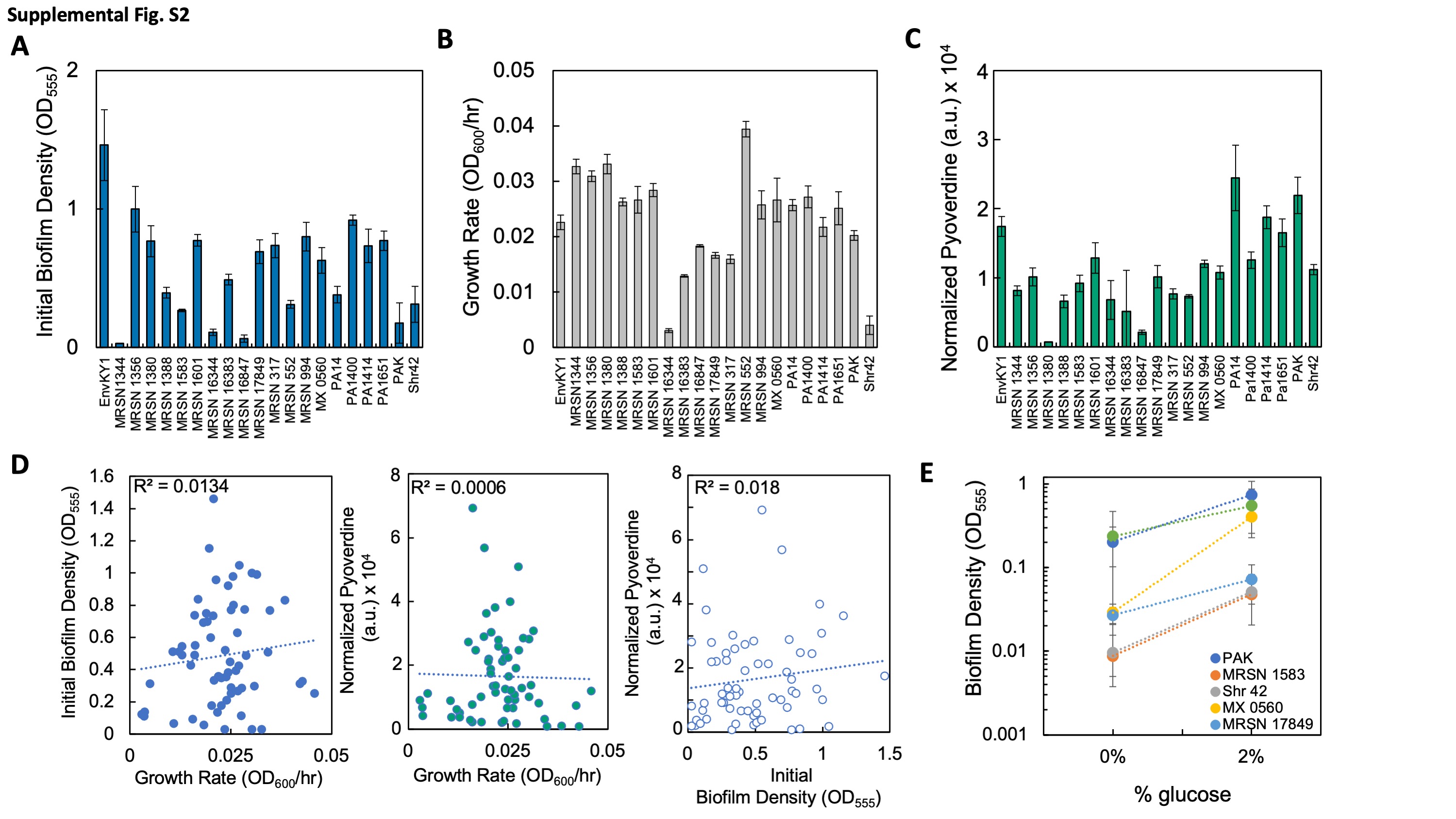

Supplement: FIG S2 [file msystems.00961-21-sf002.jpg]

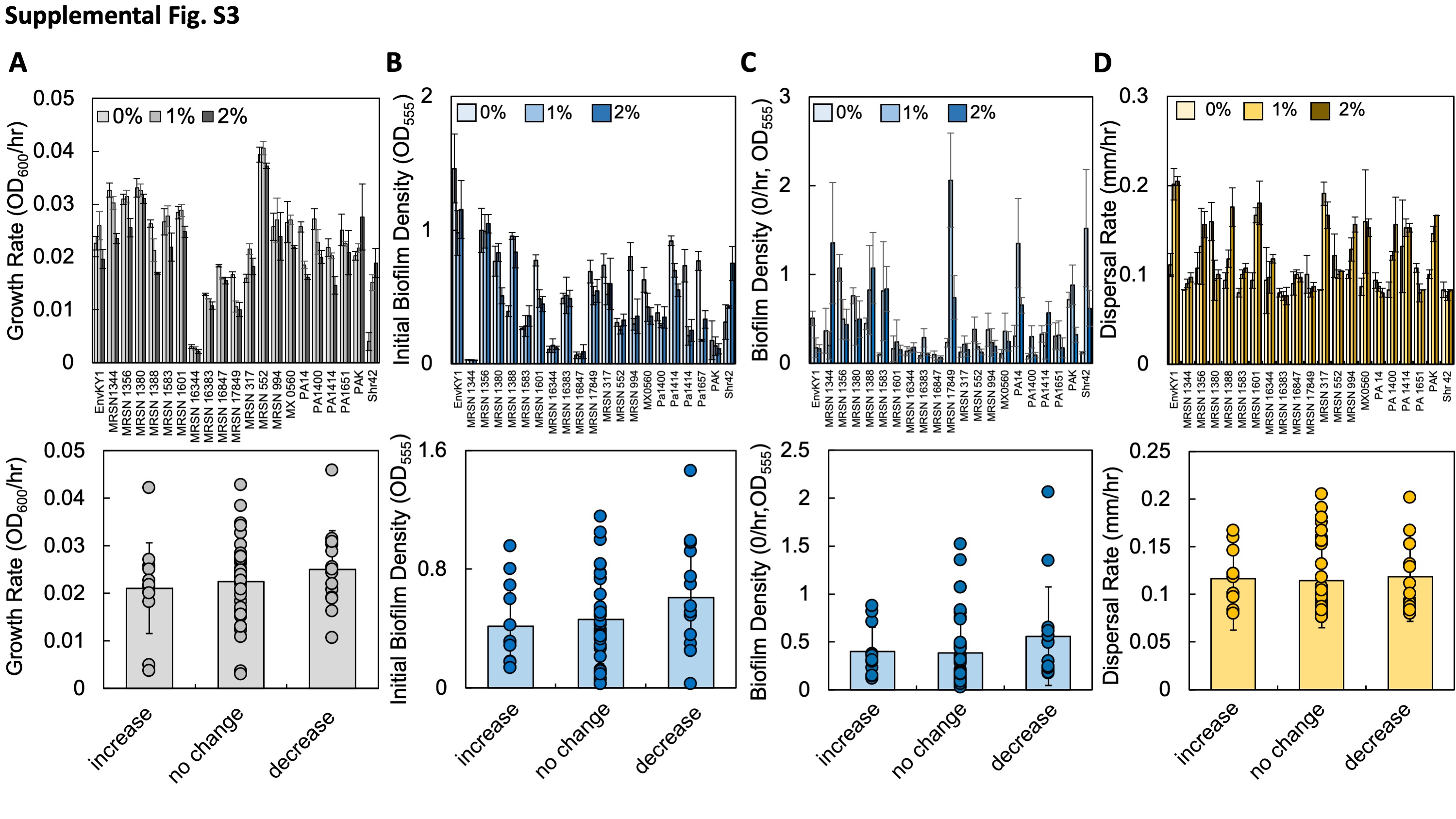

Supplement: FIG S3 [file msystems.00961-21-sf003.jpg]

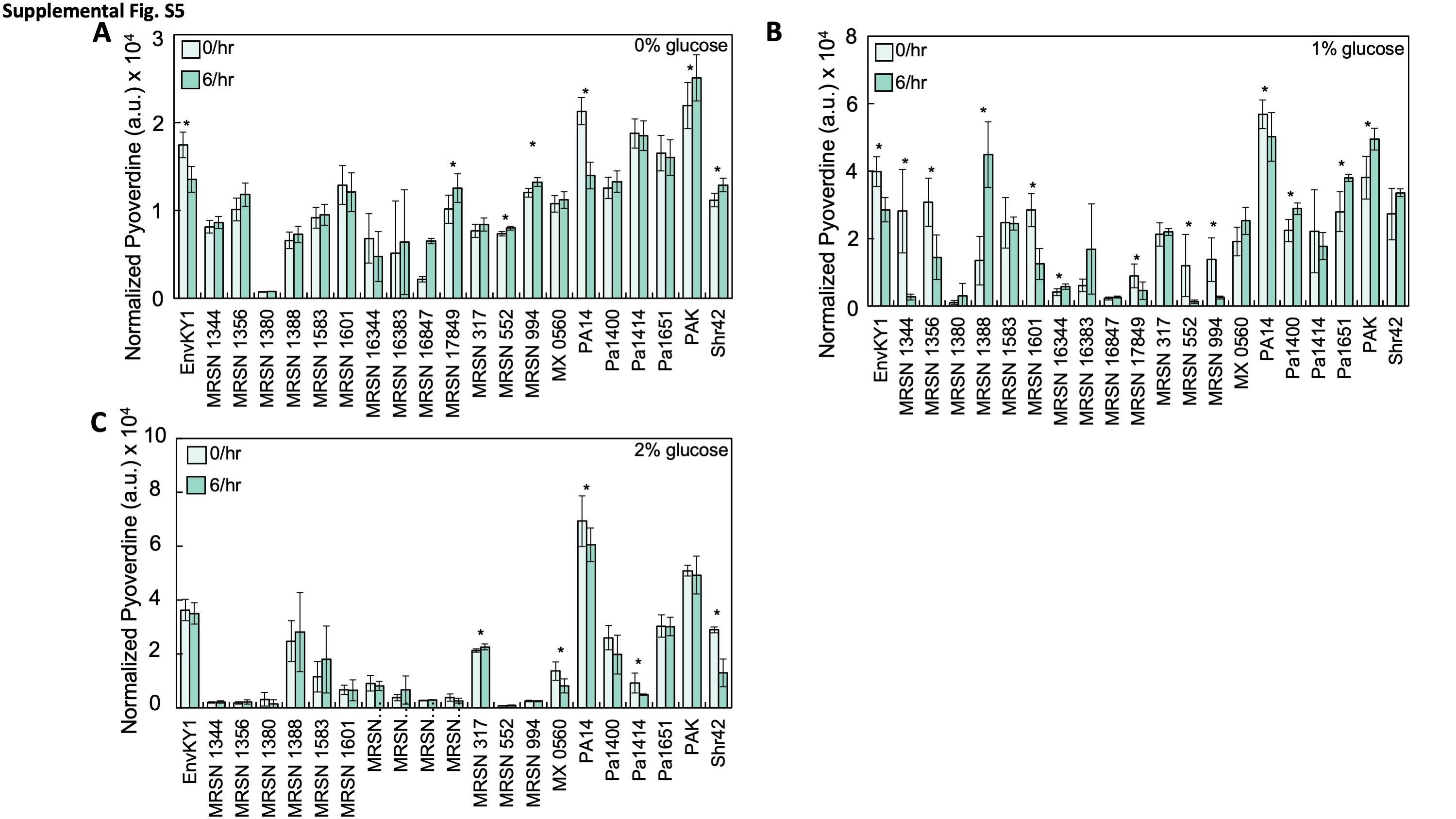

Supplement: FIG S5 [file msystems.00961-21-sf005.jpg]

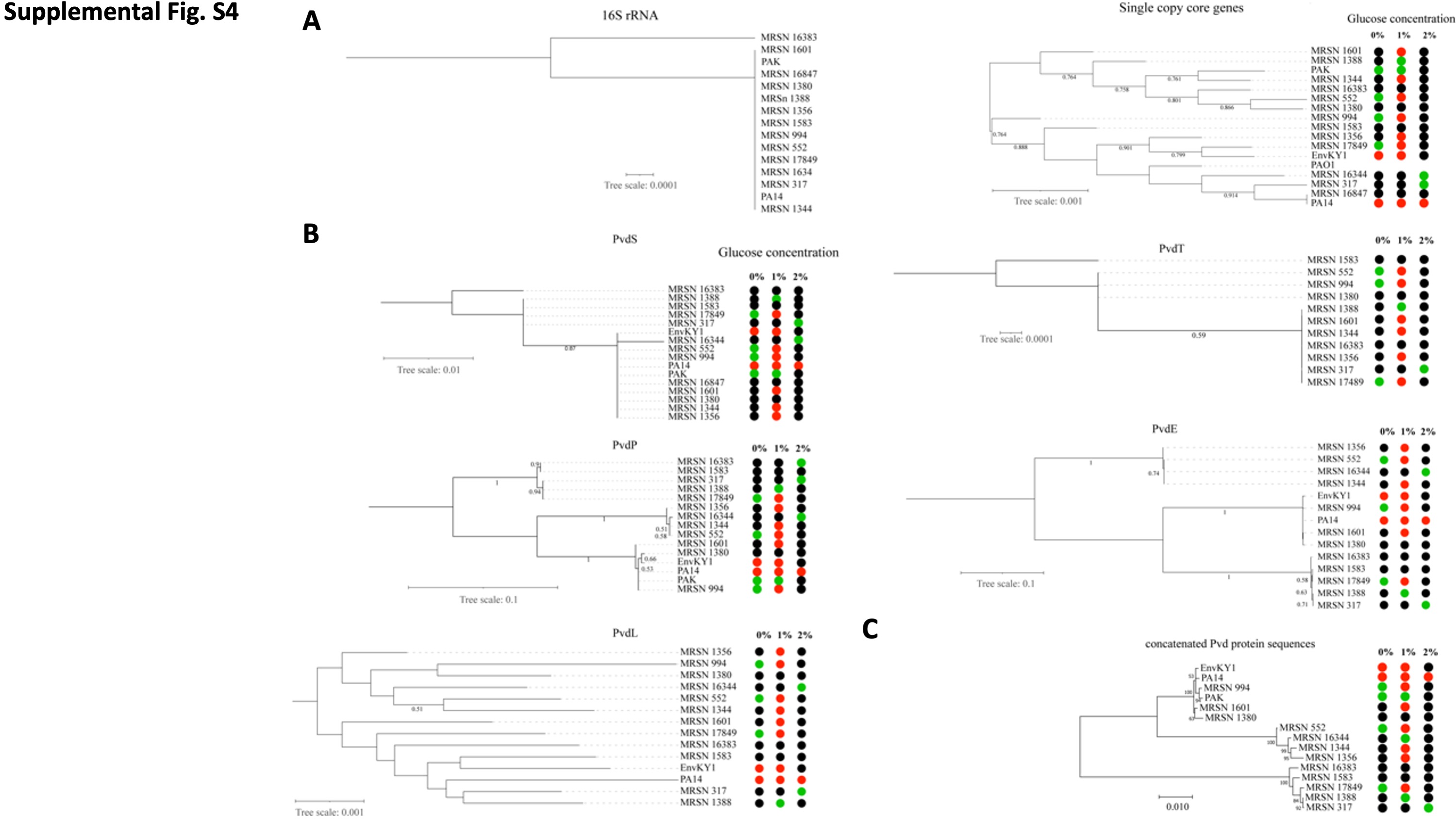

Supplement: FIG S4 [file msystems.00961-21-sf004.jpg]

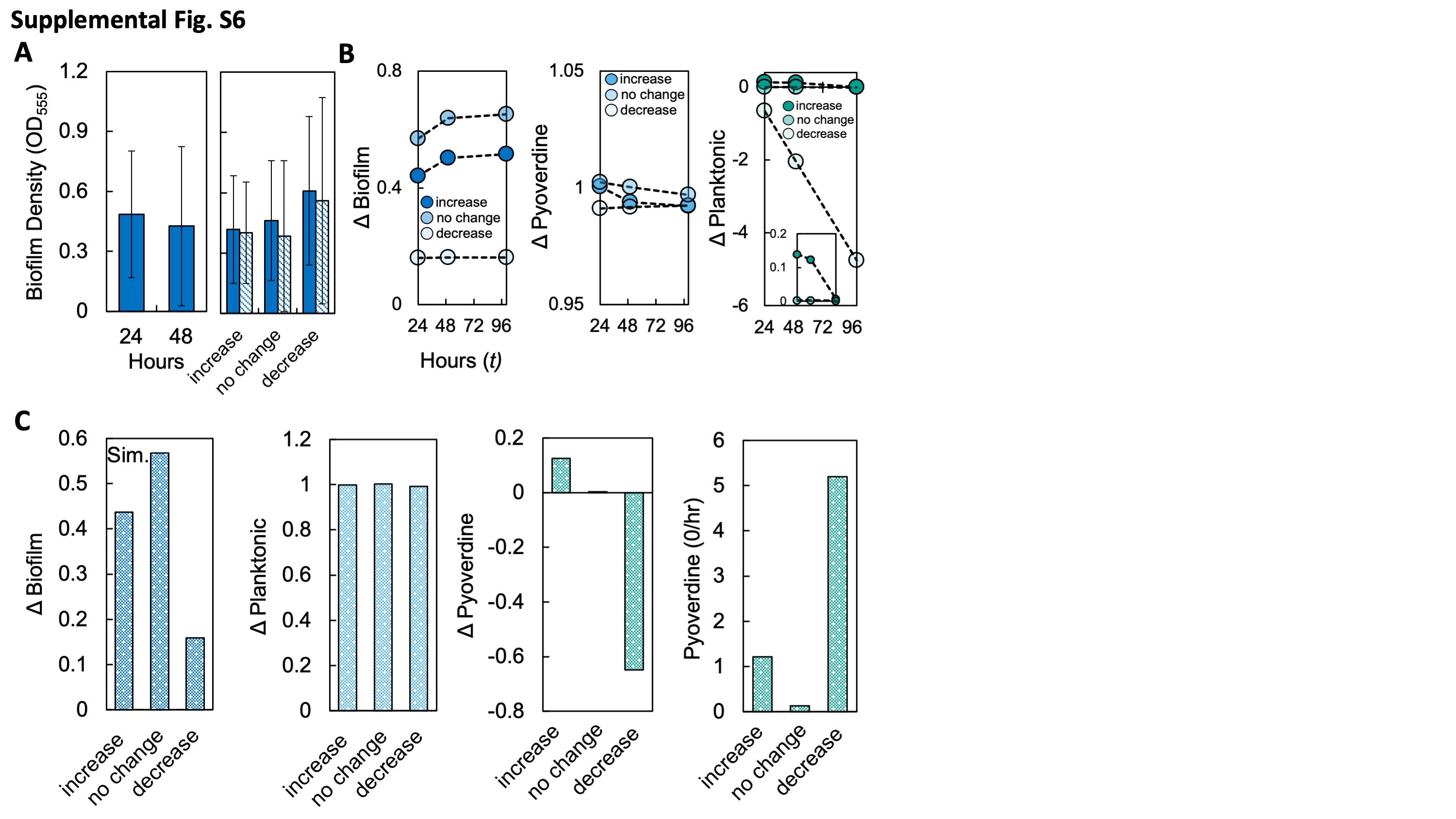

Supplement: FIG S6 [file msystems.00961-21-sf006.jpg]

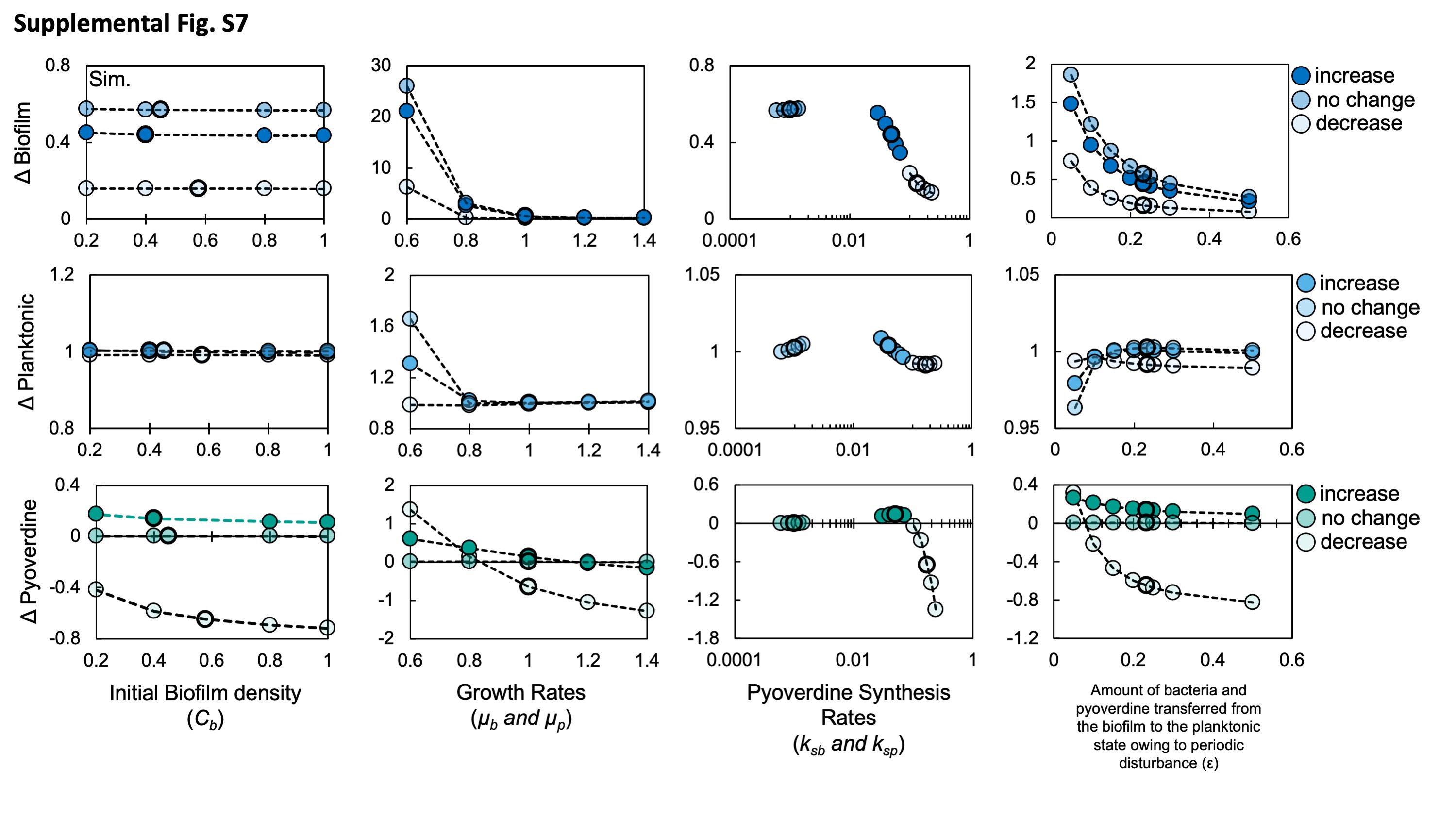

Supplement: FIG S7 [file msystems.00961-21-sf007.jpg]
