# Supplementary material for: Periodically Disturbing the Spatial Structure of Biofilms Can Affect the Production of an Essential Virulence Factor in Pseudomonas aeruginosa
Source: mSystems. 2021 Sep 28;6(5):e00961-21. doi: 10.1128/mSystems.00961-21 (PMC8547473; doi:10.1128/mSystems.00961-21)
Supplement: TABLE S3 [file msystems.00961-21-st003.docx]

**Supplemental Table S3.**

| **Parameter** | **Description** | **Value** | | **Reference** |
| --- | --- | --- | --- | --- |
| *β* | Fraction of pyoverdine that diffuses from the biofilm to the planktonic state | 0.7 | | (18) and estimated as described in Materials and Methods |
| *γ* | Fraction of bacteria that move from the biofilm to the planktonic state owing to natural processes | 0.0001 | | (19, 20) |
| *k_sb_* | Synthesis rate constant of pyoverdine for bacteria in the biofilm state | 0.17 μM/hr (high) | | (9, 10) |
|  |  | 0.05 μM/hr (int.) | |  |
|  |  | 0.001 μM/hr (low) | |  |
| *k_sp_* | Synthesis rate constant of pyoverdine for bacteria in the planktonic state | 0.20 μM/hr (high) | | (18) |
|  |  | 0.06 μM/hr (int.) | |  |
|  |  | 0.0012 μM/hr (low) | |  |
| *k_d_* | Degradation rate of pyoverdine | 0.005 /hr | | (21) |
| *μ_b_* | Growth rate of bacteria in the biofilm state | 1 /hr | | (22, 23) and estimated as described in Materials and Methods |
| *μ_p_* | Growth rate of bacteria in the planktonic state | 1.2 /hr | | (24) |
| *δ* | Maximal rate at which growth is reduced owing to lack of iron uptake via pyoverdine | 0.11 μM/hr | | Estimated as described in Materials and Methods |
| *A* | Concentration of pyoverdine that leads to half maximal growth reduction | 0.12 μM | | Estimated as described in Materials and Methods |
| *ε* | Amount of bacteria and pyoverdine transferred from the biofilm to the planktonic state owing to periodic disturbance | 0.233 | | Estimated from Fig. 1B |
| *σ* | Fraction of bacteria that are recruited from the planktonic state to the biofilm state owing to frequency dependent shear force | Fit to a polynomial. Equation of line presented in Materials and Methods. | | |
| *C_max_* | Carrying capacity of the biofilm and planktonic states | 1 | | Estimated |
| Initial [*pyo_b_*] | Initial amount of pyoverdine in the biofilm | 0.133 μM | | Estimated |
| Initial *C_b_* | Initial amount of bacteria in biofilm state | Base case/high pyo. production = 0.577 | | Estimated from Supplemental Fig. S3B |
|  |  | Int. pyo. production = 0.4 | |  |
|  |  | Low pyo. production = 0.45 | |  |
| **Parameters used when perturbing the percentage of glucose in the growth medium.** | | | | |
| **Parameter** | **Description** | **% of glucose** | **Value** | **Units** |
| *k_sb_* | synthesis rate constant of pyoverdine for bacteria in the biofilm state | 0 | 0.17 | μM/hr |
|  |  | 1 | 0.18 |  |
|  |  | 2 | 0.19 |  |
| *k_sp_* | synthesis rate constant of pyoverdine for bacteria in the planktonic state | 0 | 0.20 |  |
|  |  | 1 | 0.216 |  |
|  |  | 2 | 0.228 |  |
| *μ_b_* | growth rate of bacteria in the biofilm state | 0 | 1 | /hr |
|  |  | 1 | 0.95 |  |
|  |  | 2 | 0.9 |  |
| *μ_p_* | growth rate of bacteria in the planktonic state | 0 | 1.2 |  |
|  |  | 1 | 1.14 |  |
|  |  | 2 | 1.08 |  |
| *Initial C_b_* | initial amount of bacteria in biofilm state | 0 | 0.577 | Normalized to *C_max_* |
|  |  | 1 | 0.527 |  |
|  |  | 2 | 0.477 |  |

**Supplemental Literature Cited**

1. Arkin AP, Cottingham RW, Henry CS, Harris NL, Stevens RL, Maslov S, Dehal P, Ware D, Perez F, Canon S. 2018. KBase: the United States department of energy systems biology knowledgebase. Nature biotechnology 36:566.

2. Le KY, Otto M. 2015. Quorum-sensing regulation in staphylococci-an overview. Frontiers in microbiology 6:1174-1174.

3. Ali L, Goraya MU, Arafat Y, Ajmal M, Chen J-L, Yu D. 2017. Molecular Mechanism of Quorum-Sensing in Enterococcus faecalis: Its Role in Virulence and Therapeutic Approaches. International journal of molecular sciences 18:960.

4. Lewenza S, Conway B, Greenberg EP, Sokol PA. 1999. Quorum sensing in Burkholderia cepacia: identification of the LuxRI homologs CepRI. Journal of bacteriology 181:748-756.

5. Huber B, Riedel K, Hentzer M, Heydorn A, Gotschlich A, Givskov M, Molin S, Eberl L. 2001. The cep quorum-sensing system of Burkholderia cepacia H111 controls biofilm formation and swarming motility. Microbiology 147:2517-2528.

6. Alhede M, Bjarnsholt T, Jensen PØ, Phipps RK, Moser C, Christophersen L, Christensen LD, van Gennip M, Parsek M, Høiby N. 2009. Pseudomonas aeruginosa recognizes and responds aggressively to the presence of polymorphonuclear leukocytes. Microbiology 155:3500-3508.

7. Mizan MFR, Jahid IK, Kim M, Lee K-H, Kim TJ, Ha S-D. 2016. Variability in biofilm formation correlates with hydrophobicity and quorum sensing among Vibrio parahaemolyticus isolates from food contact surfaces and the distribution of the genes involved in biofilm formation. Biofouling 32:497-509.

8. Wang L, Ling Y, Jiang H, Qiu Y, Qiu J, Chen H, Yang R, Zhou D. 2013. AphA is required for biofilm formation, motility, and virulence in pandemic Vibrio parahaemolyticus. International journal of food microbiology 160:245-251.

9. Kang D, Turner KE, Kirienko NV. 2018. PqsA promotes pyoverdine production via biofilm formation. Pathogens 7:3.

10. Visaggio D, Pasqua M, Bonchi C, Kaever V, Visca P, Imperi F. 2015. Cell aggregation promotes pyoverdine-dependent iron uptake and virulence in Pseudomonas aeruginosa. Frontiers in microbiology 6:902.

11. Higgins DA, Pomianek ME, Kraml CM, Taylor RK, Semmelhack MF, Bassler BL. 2007. The major Vibrio cholerae autoinducer and its role in virulence factor production. Nature 450:883-886.

12. Heithoff DM, Mahan MJ. 2004. Vibrio cholerae biofilms: stuck between a rock and a hard place. Journal of bacteriology 186:4835-4837.

13. Charlebois A, Jacques M, Archambault M. 2016. Comparative transcriptomic analysis of Clostridium perfringens biofilms and planktonic cells. Avian Pathology 45:593-601.

14. Castro J, Machado D, Cerca N. 2019. Unveiling the role of Gardnerella vaginalis in polymicrobial Bacterial Vaginosis biofilms: the impact of other vaginal pathogens living as neighbors. The ISME journal 13:1306-1317.

15. Friedman L, Kolter R. 2004. Genes involved in matrix formation in Pseudomonas aeruginosa PA14 biofilms. Molecular microbiology 51:675-690.

16. Kang D, Revtovich AV, Chen Q, Shah KN, Cannon CL, Kirienko NV. 2019. Pyoverdine-Dependent Virulence of Pseudomonas aeruginosa Isolates From Cystic Fibrosis Patients. Frontiers in microbiology 10:2048.

17. Dietrich LE, Price‐Whelan A, Petersen A, Whiteley M, Newman DK. 2006. The phenazine pyocyanin is a terminal signalling factor in the quorum sensing network of Pseudomonas aeruginosa. Molecular microbiology 61:1308-1321.

18. Julou T, Mora T, Guillon L, Croquette V, Schalk IJ, Bensimon D, Desprat N. 2013. Cell–cell contacts confine public goods diffusion inside Pseudomonas aeruginosa clonal microcolonies. Proceedings of the National Academy of Sciences 110:12577-12582.

19. Sauer K, Cullen MC, Rickard AH, Zeef LAH, Davies DG, Gilbert P. 2004. Characterization of nutrient-induced dispersion in Pseudomonas aeruginosa PAO1 biofilm. Journal of bacteriology 186:7312-7326.

20. Morgan R, Kohn S, Hwang S-H, Hassett DJ, Sauer K. 2006. BdlA, a chemotaxis regulator essential for biofilm dispersion in Pseudomonas aeruginosa. Journal of bacteriology 188:7335-7343.

21. Jin Z, Li J, Ni L, Zhang R, Xia A, Jin F. 2018. Conditional privatization of a public siderophore enables Pseudomonas aeruginosa to resist cheater invasion. Nature communications 9:1-11.

22. Mah T-FC, O'Toole GA. 2001. Mechanisms of biofilm resistance to antimicrobial agents. Trends in Microbiology 9:34-39.

23. Wentland EJ, Stewart PS, Huang CT, McFeters GA. 1996. Spatial variations in growth rate within Klebsiella pneumoniae colonies and biofilm. Biotechnology progress 12:316-321.

24. Yang L, Haagensen JAJ, Jelsbak L, Johansen HK, Sternberg C, Høiby N, Molin S. 2008. In situ growth rates and biofilm development of Pseudomonas aeruginosa populations in chronic lung infections. Journal of bacteriology 190:2767-2776.
