# Supplementary material for: Periodically Disturbing the Spatial Structure of Biofilms Can Affect the Production of an Essential Virulence Factor in Pseudomonas aeruginosa
Source: mSystems. 2021 Sep 28;6(5):e00961-21. doi: 10.1128/mSystems.00961-21 (PMC8547473; doi:10.1128/mSystems.00961-21)
Supplement: TABLE S2 [file msystems.00961-21-st002.docx]

**Supplemental Table S2.**

| **Strain name** | **Source** |
| --- | --- |
| PA14 | Lingchong You |
| PA14 Δ*pel* | (15) |
| PA14 Δ*pvd* | (16) |
| PA14 *Δphz1*/*Δphz2* | (17) |
| ENKY1 | BEI Resources* |
| MRSN 1344 |  |
| MRSN 1356 |  |
| MRSN 1380 |  |
| MRSN 1388 |  |
| MRSN 1583 |  |
| MRSN 1601 |  |
| MRSN 16344 |  |
| MRSN 16383 |  |
| MRSN 16847 |  |
| MRSN 17849 |  |
| MRSN 317 |  |
| MRSN 552 |  |
| MRSN 994 |  |
| MX0560 |  |
| PA1400 |  |
| PA1414 |  |
| PA1651 |  |
| PAK |  |
| Shr42 |  |

*MRSN indicates that the strain was ultimately obtained from the Multidrug-Resistant Organism Repository and Surveillance Network (MRSN) at the Walter Reed Army Institute of Research (WRAIR), and distributed by BEI Resources.
